# A Combination of Deworming and Prime-Boost Vaccination Regimen Restores Efficacy of Vaccination Against Influenza in Helminth-Infected Mice

**DOI:** 10.3389/fimmu.2021.784141

**Published:** 2021-12-21

**Authors:** Nadine Stetter, Wiebke Hartmann, Marie-Luise Brunn, Stephanie Stanelle-Bertram, Gülsah Gabriel, Minka Breloer

**Affiliations:** ^1^ Section for Molecular Biology and Immunology, Helminth-Immunology Group, Bernhard Nocht Institute for Tropical Medicine, Hamburg, Germany; ^2^ Research Department for Viral Zoonoses - One Health, Leibniz Institute for Experimental Virology Heinrich Pette Institute (HPI), Hamburg, Germany; ^3^ Institute for Virology, University for Veterinary Medicine Hannover, Hannover, Germany; ^4^ Department for Biology, University Hamburg, Hamburg, Germany

**Keywords:** anthelmintic treatment, flubendazole, immunomodulation, influenza, *Litomosoides sigmodontis*, parasite infection, prime-boost, vaccination efficacy

## Abstract

Helminths still infect a quarter of the human population. They manage to establish chronic infections by downmodulating the immune system of their hosts. Consequently, the immune response of helminth-infected individuals to vaccinations may be impaired as well. Here we study the impact of helminth-induced immunomodulation on vaccination efficacy in the mouse system. We have previously shown that an underlying *Litomosoides sigmodontis* infection reduced the antibody (Ab) response to anti-influenza vaccination in the context of a systemic expansion of type 1 regulatory T cells (Tr1). Most important, vaccine-induced protection from a challenge infection with the 2009 pandemic H1N1 influenza A virus (2009 pH1N1) was impaired in vaccinated, *L. sigmodontis-*infected mice. Here, we aim at the restoration of vaccination efficacy by drug-induced deworming. Treatment of mice with Flubendazole (FBZ) resulted in elimination of viable *L. sigmodontis* parasites in the thoracic cavity after two weeks. Simultaneous FBZ-treatment and vaccination did not restore Ab responses or protection in *L. sigmodontis-*infected mice. Likewise, FBZ-treatment two weeks prior to vaccination did not significantly elevate the influenza-specific Ig response and did not protect mice from a challenge infection with 2009 pH1N1. Analysis of the regulatory T cell compartment revealed that *L. sigmodontis-*infected and FBZ-treated mice still displayed expanded Tr1 cell populations that may contribute to the sustained suppression of vaccination responses in successfully dewormed mice. To outcompete this sustained immunomodulation in formerly helminth-infected mice, we finally combined the drug-induced deworming with an improved vaccination regimen. Two injections with the non-adjuvanted anti-influenza vaccine Begripal conferred 60% protection while MF59-adjuvanted Fluad conferred 100% protection from a 2009 pH1N1 infection in FBZ-treated, formerly *L. sigmodontis-*infected mice. Of note, applying this improved prime-boost regimen did not restore protection in untreated *L. sigmodontis-*infected mice. In summary our findings highlight the risk of failed vaccinations due to helminth infection.

## Introduction

More than a quarter of the human population suffers from helminth infections (1, 2). These large multicellular parasites manage to survive within their hosts by actively downmodulating the immune response that is directed towards themselves (3, 4). Thereby helminth-induced immunomodulation is not restricted to anti-helminth immunity but “spills over” to unrelated antigens. Accordingly, the immune response of a helminth-infected individual to co-infecting pathogens or to a vaccination may be compromised. Several human studies suggest that an underlying helminth infection interfere with vaccination efficacy (5–9). While most studies demonstrate reduced cellular and humoral vaccination responses in the helminth-infected population compared to an uninfected endemic control group, the efficacy of vaccination in terms of vaccination-induced protection from a challenge infection is difficult to test in human studies.

We investigate the impact of concurrent helminth infection on the outcome of vaccination in the controlled setting of the mouse system using *Litomosoides sigmodontis* as an established model for human filarial infections (10–12). *L. sigmodontis* is a natural parasite of cotton rats (*Sigmodon hispidus*) that successfully infects several laboratory mouse strains (11, 12). Infective 3^rd^ stage larvae (L3) are transmitted *via* the blood meal by the intermediate host, the mite *Ornithonyssus bacoti*, to the definitive host. The transmitted L3 migrate *via* the lymphatics to the thoracic cavity by day 4 post infection (p.i.) and moult *via* a 4^th^ larval stage (L4) to parasitic adults by day 30. Semi-susceptible C57BL/6 mice eliminate these worms *via* granuloma formation from day 35 onwards before sexual maturity is reached, while fully susceptible BALB/c mice stay infected for more than 3 months and allow reproduction and production of 1^st^ stage larvae or microfilariae (10, 13, 14).

Using this model, we showed previously that an underlying *L. sigmodontis* infection reduced the efficacy of a CD8^+^ T cell inducing protein-based malaria vaccination, causing impaired protection against a *Plasmodium berghei* sporozoite challenge infection in vaccinated *L. sigmodontis-*infected mice compared to non-helminth-infected mice (15). Likewise, the proliferation of model-antigen-specific CD4^+^ T cells and the antibody (Ab) response to thymus-dependent antigens that require the activation of follicular T helper cells (T_FH_) were suppressed in infected mice compared to non-helminth-infected mice (16–18). Of note, this suppression was observed in *L. sigmodontis-*infected fully susceptible BALB/c mice and in semi-susceptible C57BL/6 mice to the same extent (15–18).

To test the clinical relevance of the reduced Ab response, we further investigated the outcome of vaccination against influenza in *L. sigmodontis-*infected mice. Seasonal influenza causes up to half a million deaths worldwide every year and can be prevented by vaccinations that induce a neutralizing Ab response to the hemagglutinin (HA) head of the virus (19, 20). Using the trivalent split subunit vaccine Begripal that is licensed for humans, we reported that neutralizing as well as HA-specific IgG responses were reduced in mice that carried an *L. sigmodontis-*infection at the moment of vaccination (21). A challenge infection of C57BL/6 mice with the human 2009 pandemic H1N1 influenza A virus strain A/Hamburg/NY1580/09 (2009 pH1N1) resulted in virus replication in the lung that was accompanied by transient weight loss (22–24). Vaccination with Begripal protected non-helminth-infected mice from 2009 pH1N1 challenge infection-induced weight loss and reduced the viral load while vaccinated *L. sigmodontis-*infected mice displayed high viral burden and replicating influenza virus in the lungs (21). This suppression was observed in mice that carried living parasites but also in mice that were vaccinated after immune-driven termination of the *L. sigmodontis* infection (17, 21).

Here, we attempte to restore vaccination efficacy by drug-induced deworming. We report that efficient deworming simultaneous with influenza vaccination or two weeks before influenza vaccination did not establish protection from a 2009 pH1N1 challenge infection in *L. sigmodontis-*infected mice. Likewise, the introduction of a prime-boost vaccination regimen with either Begripal, or the MF59-adjuvanted influenza vaccine Fluad did not rescue vaccination efficacy in *L. sigmodontis-*infected mice. However, a combination of drug-induced deworming and improved vaccination regimens outcompeted the helminth-induced immunomodulation and led to sterile immunity in formerly *L. sigmodontis-*infected mice.

## Materials and Methods

### Ethics Statement

All animal experimentations were conducted at the specific pathogen-free animal facility of the Bernhard Nocht Institute for Tropical Medicine (BNITM) in agreement with the German Animal Welfare Act and the relevant German authority (Behörde für Gesundheit und Verbraucherschutz, Hamburg, approval numbers 84/15, 103/2018. All mice were kept in individually ventilated cages (maximum of 5 mice per cage). Mice were sacrificed by an overdosed CO_2_ narcosis followed by cervical dislocation in accordance with the German animal protection law. 8-12 week old C57BL/6 mice were used and either bred in the animal facility of the BNITM or were obtained from Janvier Labs.

### Life Cycle and *L. sigmodontis* Infection

The life cycle of *L. sigmodontis* was maintained in their natural reservoir, the cotton rats (*Sigmodon hispidus*). Therefore, cotton rats were anesthetized by isoflurane narcosis and blood was collected from the retro-bulbar sinus in order to count the microfilariae (MF, L1). Cotton rats, which were used for further infection of blood-sucking mites (*Ornithonyssus bacoti*), had an infection rate of 500 – 2000 MF per µl blood. Infected cotton rates were exposed to mites that ingested MF during a blood meal. Infected mites were kept at 29°C and 90% humidity for 14 days to allow maturation of L1 to L3. Experimental mice were anesthetized with ketamine/xylazine (100 mg and 5 mg/kg body weight) and exposed to these infected mites for 16 hours, i.e., naturally infected. To quantify the worm load, infected mice were sacrificed at indicated time points and the thoracic cavity was flushed with 10 mL PBS. Viable worms were defined by movement. Coated worms were defined as worms either moving or immobile that were covered with host cells. By contrast, dead worms were free of visible host cells and did not move anymore. The anthelminthic drug flubendazole (FBZ, Sigma, Germany) was administered by repeated s.c. injection on 5 consecutive days with a dose of 5 mg/kg body weight.

### Influenza Virus Infection

For influenza infection C57BL/6 mice were anesthetized with ketamine/xylazine (100 mg and 5 mg/kg body weight) and i.n. infected with 25 µL 1 x 10^3^ plaque forming units (PFU) 2009 pH1N1 influenza A Hamburg/NY1580/09. The influenza virus was isolated from pharyngeal swabs of a female patient as described previously (23). Health status of the mice was monitored daily according to the animal protocols approved by the Hamburg authorities until day 3 post challenge infection when mice were sacrificed to quantify viral burden in the lungs.

### Vaccination and Quantification of the Vaccine-Specific Humoral Response

Mice were vaccinated by i.p. injection of 3.75 µg either non-adjuvanted (Begripal, Seqirus) or adjuvanted (Fluad, Seqirus) vaccine against influenza in 200 µL PBS (Carl Roth, Germany). Blood was collected from the vena fascialis at the indicated time points, 2-4 weeks after vaccination, and allowed to coagulate for 1 h at RT. After centrifugation (10.000 x g for 10 min) serum was transferred into a fresh tube and stored at -20°C until further analysis.

### Hemagglutinin Inhibition (HI) Assay

Murine serum samples were thawed and heat inactivated for 30 min at 56°C in a water bath (Memmert, Germany). Serial dilutions of sera in 25 µL PBS (Carl Roth, Germany) were incubated with 25 µl of 2009 pH1N1 influenza A virus in duplicates in 96-well-V-plates (Greiner Bio-one, Austria). The virus solution was standardized to 8 hemagglutination units before. After 30 min incubation at room temperature, 50 µL 1% fresh chicken red blood cell solution (Lohman, Germany) in 0.9% NaCl (Carl Roth, Germany) was added to each well. The dilution that still inhibited agglutination was calculated as a titre after a further 1-hour-incubation at 4°C.

### Cultivation of MDCK Cells Prior to the Plaque Assay

Madin-Darby canine kidney (MDCK) cells were provided by G. Gabriel (Heinrich-Pette Institute, Hamburg, Germany). Cells were stored in liquid nitrogen and thawed 2 weeks prior to the plaque assay. Cells were quickly transferred to pre-warmed RPMI 1640 medium with L-Glutamine (Lonza, Switzerland) supplemented with 10% FCS (Capricorn Scientific, Germany) and washed twice. Cells were grown in T75 flasks (Greiner Bio-one, Austria) for 2 weeks in 15 ml RPMI 1640 medium with L-Glutamine supplemented with 5% FCS, Hepes (Lonza, Switzerland), and Gentamycin (Lonza, Switzerland). Cells were splitted every 3-4 days in a 1:6 ratio. One day prior to the start of plaque assay, cells from one confluent T75 were splitted to 4-5 6-Well plates (Greiner Bio-one, Austria). MDCK cells were grown overnight at 37°C in 3 ml RPMI cell culture medium which results in a semi-confluent cell layer.

### Detection of Viral Loads in the Lungs

Viral loads were determined in lung homogenates by MDCK plaque assay. Lungs were removed at indicated time points and stored at -70°C in 0.1% BSA (Serva, Germany) in PBS. Lungs were thawed and homogenized. The supernatant was collected after centrifugation (10 min, 950 x g, 4°C). Serial dilutions of lung homogenates (10-^1^ to 10^-6^) were added to a confluent MDCK cell culture in 6-well-plates. After 30 min at 37°C, inoculum was removed, cells washed one time with 1x PBS and overlaid or plates were directly overlaid with 3 mL 1.25% Avicel (FMC, USA) in Minimum Essentiel Medium (Lonza, Switzerland) containing 1µg/mL N-tosyl-L-phenylalanine chloromethyl ketone (TPCK) treated -Trypsin (Sigma, Germany). After a further incubation for 72 h at 37°C and 5% CO_2_, the overlay was removed and the plates were washed with PBS. The cells were fixed with 0.5 mL 4% paraformaldehyde (PFA, Carl Roth, Germany) for 30 min at 4°C. PFA was removed and plates were incubated for 10 min with 1 mL/well 1% crystal violet (Merk, Germany). The staining was stopped by removing crystal violet and washing the plates with tap water.

### Isolation of Cells From the Spleen and the Thoracic Cavity

The thoracic cavity was flushed with 10 ml cold PBS. Cells were sedimented by centrifugation (1200 rpm, 4°C, 6 min) and the number of cells determined by counting with trypan blue (Sigma, Germany). Spleens were removed and transferred to a 50 ml tube with 10 ml PBS and kept on ice until further processing. Spleens were pounded between two glass slides (Engelbrecht, Germany) in a petri dish (Sarstedt, Germany) and transferred back to the 50 ml tube. After centrifugation (1200 rpm, 4°C, 6 min), the supernatant was discarded and erythrocytes lysed by addition of 5 ml ACK lysis buffer (150 mM NH_4_Cl, 1 mM KHCO_3_, 0.1 mM Na_2_EDTA) for 2 min at room temperature. Lysis was stopped by addition of 30 ml cold PBS. Cells were centrifuged again and the supernatant discarded. The cell pellet was resuspended in 10 ml PBS and filtered over a 70 µm Falcon™ cell strainer (Corning). The cell strainer was washed with 10 ml PBS and the cell suspension centrifuged. The obtained cell pellet was resuspended in 10 ml PBS and counted in a 1:10 dilution with trypan blue. To obtain single cell suspension cells were vortexed during washing steps. Single cells were confirmed by trypan blue (Sigma, Germany) cell counting under a microscope.

### Flow Cytometry

Single cells (3 x 10^6^) from the spleen or thoracic cavity (TC) were stained with 1 µL Zombie UV™ Fixable Viability Kit or Zombie Aqua Fixable Viability Kit in 1 mL PBS for 30 min at 4°C. For surface staining, cells were first stained for 15 min at 37°C with APC-labelled anti-mouse LAG-3 (clone: C9b7W, Lot: B215463) and PE/Cy7-labeled anti-mouse CD49b (clone: Hma2, Lot: B210811) Ab. After an additional incubation for 15 min at RT, cells were washed and stained for 30 min at 4°C with BV510-labeled anti-mouse CD4 (clone: RM4-5, Lot: B277537). For subsequent intracellular staining with anti-mouse AF700-labeled anti-mouse Foxp3 (clone: FJK-16S, Lot: 4307313), cells were fixed and permeabilized with Thermofisher Scientific Foxp3/Transcription factor staining buffer set (Thermofisher, Germany) according to the manufacturer’s protocol. Samples were analysed on a LSRII (Becton Dickinson) using FlowJo software (TreeStar).

### Statistical Analysis

Data were analysed using Graph Pad Prism, tested for normality distribution and further tested either with unpaired t test (parametric) or Mann-Whitney test (non-parametric). Asterisks for all analyses * p ≤ 0.05, ** p ≤ 0.01, *** p ≤ 0.001, **** p ≤ 0.0001.

## Results

### Drug-Induced Deworming Does Not Restore Influenza Vaccination-Induced Protection in *L. sigmodontis-*Infected Mice

**Figure 1:**
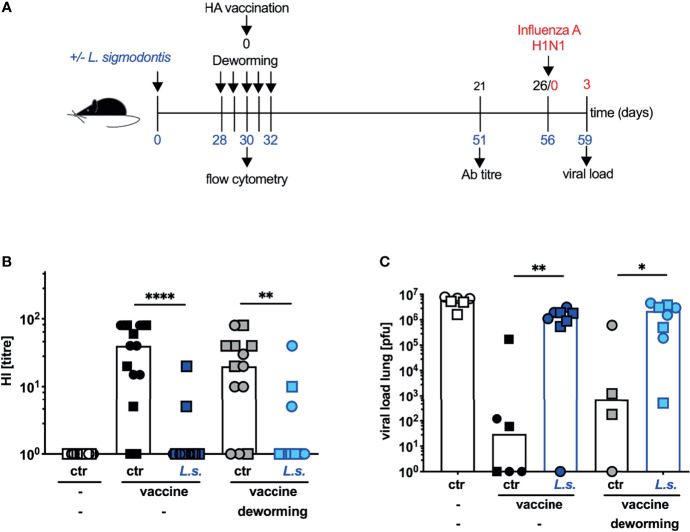
Deworming at the moment of vaccination does not restore the efficacy of vaccination against influenza. **(A)** Experimental design: Blue numbers indicate time post *L. sigmodontis* infection, black numbers indicate time post deworming and red numbers indicate time post 2009 pH1N1 influenza A virus challenge infection. C57BL/6 mice were left naive (white, black and grey symbols) or naturally infected with *L. sigmodontis* (dark and light blue symbols). Mice were dewormed by daily s.c. FBZ injection for 5 consecutive days on day 28 p.i. until day 32 p.i. (grey and light blue symbols) or were left untreated. Deworming efficacy is shown in **Supplementary Figure S1**. Mice were i.p. vaccinated with non-adjuvanted influenza vaccine Begripal at day 30 post *L. sigmodontis* infection or were left unvaccinated (white symbols). **(B)** HI titres were quantified 21 days after vaccination. **(C)** All mice were i.n. infected with 1 x 10^3^ PFU 2009 pH1N1 influenza A virus 5 days later. Viral load in the lungs was quantified day 3 post challenge infection by MDCK plaque assay. Shown are combined results out of 2 independent experiments with **(B)** n ≥ 6 and **(C)** n ≥ 3 (2 for the helminth-free and FBZ-treated group) per group and experiment. Each symbol represents an individual mouse, squares and circles represent independent experiments, the bars show the median and asterisks indicate statistically significant differences applying Mann-Whitney test (*p ≤ 0.05, **p ≤ 0.01, ****p ≤ 0.0001). (*L. sigmodontis*, *Litomosoides sigmodontis*; s.c., subcutaneous; FBZ, Flubendazole; p.i., post infection; i.p., intraperitoneal; HI, hemagglutination inhibition; i.n., intra nasal; PFU, plaque forming units; MDCK, Madin-Darby canine kidney).

We use mice that are infected with the filarial parasite *L. sigmodontis* mice as an established model for chronic human filarial infections (10–12) to study the impact of concurrent helminth infection on the efficacy of vaccinations in the mouse system. We reported previously that the efficacy of vaccination against seasonal influenza using the trivalent split subunit vaccine Begripal that is licensed for humans was impaired in mice with underlying *L. sigmodontis* infection (21). Here, we aimed at the restoration of vaccination efficacy by drug-induced deworming. To this end, mice were naturally infected with *L. sigmodontis* by exposure to infected mites. 4 weeks later, when immature adults had established an infection in the thoracic cavity, mice were treated by s.c. injection of FBZ on 5 consecutive days (**Figure 1A**). In our first approach, the anti-influenza vaccination was applied simultaneously with the deworming regimen and neutralizing Ab titres were quantified 3 weeks after vaccination by haemagglutination inhibition (HI) assay. *L. sigmodontis*-infected mice responded to the vaccination with drastically reduced HI titres compared to naïve control mice (dark blue symbols to black symbols, **Figure 1B**) as we had shown before (21). FBZ-treatment did not restore the Ab response in these helminth-infected mice (light blue symbols to grey symbols, **Figure 1B**). All mice were subsequently challenged by infection with the human pathogenic 2009 pH1N1 Influenza A strain that replicates in mice without further adaptation (22–24). Viral load in the lung was quantified three days post influenza virus infection (**Figure 1C**). Vaccinated control mice displayed reduced viral load compared to both, non-vaccinated mice and vaccinated helminth-infected mice, as expected (21). The FBZ treatment of vaccinated helminth-infected mice did not improve protection from the influenza virus challenge infection. Vaccinated FBZ-treated and helminth-infected mice displayed the same elevated viral load as the vaccinated non-treated helminth-infected mice (light blue to dark blue symbols, **Figure 1C**). As additional control, we show that FBZ-treatment of the non-helminth-infected control group did neither modulate Ab response nor 2009 pH1N1 viral load in the lung (black symbols to grey symbols p = 0.43, **Figure 1B** and p = 0.27 **Figure 1C**).

As the FBZ-treatment had no effect on responsiveness to vaccination we next controlled the efficacy of FBZ-induced deworming. To quantify the worm burden at the moment of vaccination, we recorded the number of worms in the thoracic cavity of helminth-infected FBZ-treated and untreated mice after the 3^rd^ FBZ application (**Supplementary Figure S1A**). Additionally, we recorded the worm burden 16 days after the final FBZ application to analyse the endpoint of the applied deworming regimen (**Supplementary Figure S1B**). While the worm burden was not statistically significantly reduced (p=0.07) after the third FBZ injection (**Supplementary Figure S1A**), the FBZ treatment resulted in complete clearance of viable worms and a significant reduction of killed and coated worms two weeks later (**Supplementary Figure S1B**). It should be noted that we had to shift the onset of FBZ-treatment in our second approach from day 28 to day 14 post *L. sigmodontis* infection, to allow analysis of the worm burden in FBZ-treated and untreated mice at days 30-35. An analysis at later time points would not report FBZ-mediated clearance correctly, since C57BL/6 mice start to naturally clear the *L. sigmodontis* infection *via* granuloma formation after day 35 (10, 13, 14).

In summary these results show that FBZ-treatment successfully eliminated *L. sigmodontis* parasites in the thoracic cavity of infected mice 2 weeks after the onset of therapy. Application of the vaccination against influenza to helminth*-*infected mice during the FBZ-treatment, when viable worms were still present, failed to induce a neutralizing Ab response. Subsequent protection from a 2009 pH1N1 influenza A virus challenge infection was not established.

**Figure 2:**
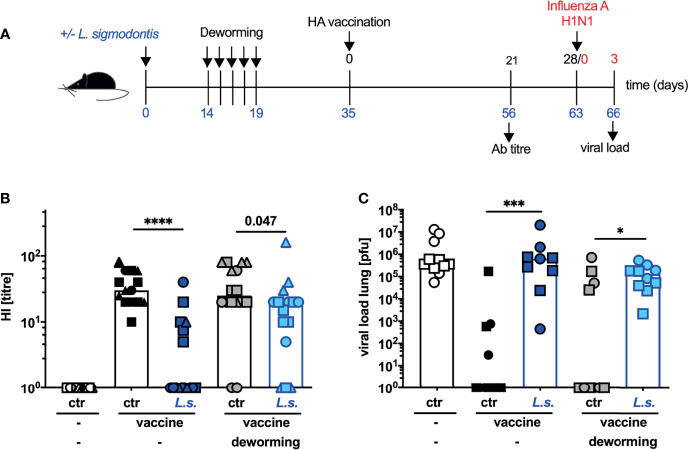
Deworming 2 weeks before vaccination against influenza results in partial restoration of neutralizing Ab response but does not confer protection. **(A)** Experimental design: C57BL/6 mice were left naive (white, black and grey symbols) or naturally infected with *L. sigmodontis* (dark and light blue symbols). Mice were dewormed by daily s.c. FBZ injection FBZ (grey and light blue symbols) for 5 consecutive days on day 14 p.i. until day 19 p.i. or were left untreated. Deworming efficacy is shown in **Supplementary Figure S1**. Mice were i.p. vaccinated with non-adjuvanted influenza vaccine Begripal at day 35 post *L. sigmodontis* infection or were left unvaccinated (white symbols). **(B)** HI titres were quantified 21 days after vaccination. **(C)** All mice were i.n. infected with 1 x 10^3^ PFU 2009 pH1N1 influenza A virus 7 days later. Influenza virus burden in the lungs were quantified day 3 post challenge infection. Shown are combined results from 3 independent experiments with n ≥ 3 **(B)** per group and experiment or combined results from 2 independent experiments with n ≥ 5 **(C)** per group and experiment. Each symbol represents an individual mouse, squares, circles and triangles represent independent experiments, the bars show the median. Asterisks indicate statistically significant differences applying Mann-Whitney test (*p ≤ 0.05, ***p ≤ 0.001, ****p ≤ 0.0001, numbers indicate *p*-values). (*L. sigmodontis*, *Litomosoides sigmodontis*; s.c., subcutaneous; FBZ, Flubendazole; p.i., post infection; i.p., intraperitoneal; HI, hemagglutination inhibition; i.n., intra nasal; PFU, plaque forming units).

Consequently, we next applied the vaccination against influenza two weeks after the final FBZ treatment, a time point when viable worms are eradicated due to drug treatment (**Supplementary Figure S1B** and **Figure 2**). The HI titre of helminth-infected and FBZ-treated mice recorded 3 weeks after the vaccination was still significantly lower than in the naive FBZ-treated control group (**Figure 2B**: p = 0.047). However, in direct comparison the helminth-infected and non-treated mice displayed a more drastic reduction in the HI titre (p < 0.0001) compared to non-helminth-infected control mice. Nevertheless, FBZ-treated helminth-infected mice were not protected against challenge infection with 2009 pH1N1 influenza A virus (**Figure 2C**). All mice that were vaccinated after FBZ-mediated clearance of the helminth infection displayed high viral loads in the lung, while the vaccinated control mice displayed sterile immunity in 7 out of 11 mice (non-helminth-infected control) and 6 out of 10 mice (non-helminth-infected and FBZ-treated control, **Figure 2C**). Again, FBZ-treatment of vaccinated, non-helminth-infected mice did not modulate HI response or viral load compared to non-treated, vaccinated non-helminth-infected mice (**Figure 2B**, black symbols to grey symbols p = 0.48; **Figure 2C**: p = 0.57).

### Drug-Induced Deworming Does Not Prevent or Revert Tr1 Cell Expansion in *L. sigmodontis-*Infected Mice

**Figure 3:**
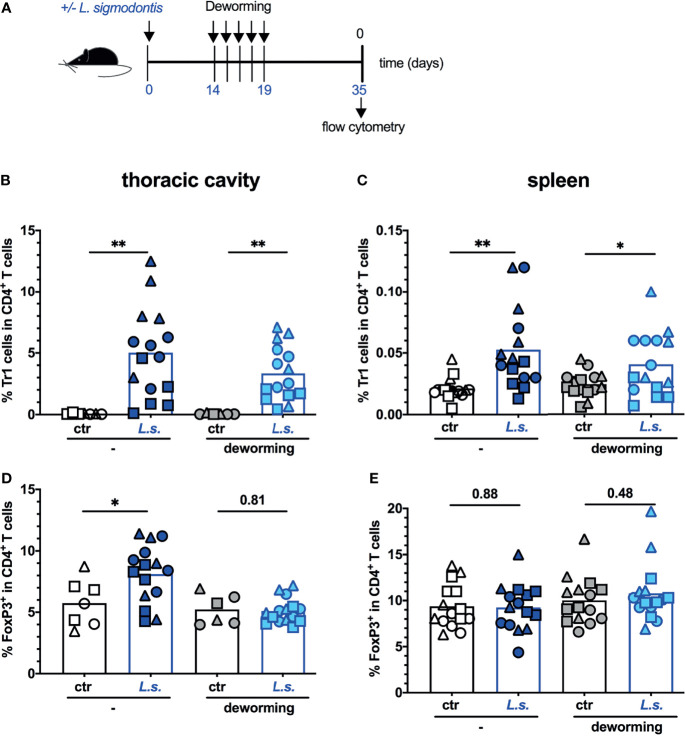
Tr1 cells remain expanded 2 weeks after deworming. **(A)** Experimental design: C57BL/6 mice were left naive (white and grey symbols) or naturally infected with *L. sigmodontis* (dark and light blue symbols). Mice were dewormed by daily s.c. FBZ injection (grey and light blue symbols) for 5 consecutive days on day 14 p.i. until day 19 p.i. or were left untreated. Deworming efficacy is shown in **Supplementary Figure S1**. Mice were sacrificed at day 35 post *L. sigmodontis* infection. Tr1 cells in thoracic cavity **(B)** or spleen **(C)** were quantified as CD4^+^Foxp3^-^LAG-3^+^CD49b^+^ cells. Treg in thoracic cavity **(D)** or spleen **(E)** were quantified as CD4^+^Foxp3^+^ cells. The gating strategy and exemplary dot plots are shown in **Supplementary Figure S2**. All original dot plots are provided in the supplementary material. Data is presented as combined results from 2-3 independent experiments with n ≥ 3 per group and experiment. Each symbol represents an individual mouse, squares, circles and triangles represent independent experiments, the bars show the mean. Asterisks indicate statistically significant differences applying unpaired t test (*p ≤ 0.05, **p ≤ 0.01, and numbers indicate *p*-values). (*L. sigmodontis*, *Litomosoides sigmodontis*; s.c., subcutaneous; FBZ, Flubendazole; p.i., post infection; Tr1, type 1 regulatory T cells; Treg, regulatory T cells; Foxp3, Forkhead-Box-Protein P3; CD, cluster of differentiation).

We have shown previously that *L. sigmodontis* infection induced a systemic and sustained expansion of type 1 regulatory T cells (Tr1) that was correlated to the observed interference with vaccination efficacy (21). To understand why the vaccination against influenza failed in the FBZ-treated previously helminth-infected mice despite the successful deworming at the time point of vaccination, we next analysed the composition of regulatory T cell populations. To this end, mice were naturally infected with *L. sigmodontis* and FBZ-treated as described in **Figure 2**. Instead of vaccinating the mice two weeks after the final FBZ treatment they were sacrificed for analysis (**Figure 3A**). Thereby, we analysed the thoracic cavity that is the site of worm infection as an indicator of local changes (**Figures 3B, D**) and the spleen as an indicator of systemic changes (**Figures 3C, E**) in the regulatory T cell populations. Foxp3^+^ regulatory T cells (Treg) were identified as CD4^+^Foxp3^+^ cells and Tr1 cells were identified as CD4^+^Foxp3^-^LAG-3^+^CD49b^+^ cells (**Supplementary Figure S2**). Helminth-infected mice displayed an elevation in Tr1 cells in the thoracic cavity (**Figure 3B**) and in the spleen (**Figure 3C**), while Treg expanded only locally in the thoracic cavity (**Figures 3D, E**), as we have shown before (21). FBZ treatment of helminth-infected mice abrogated the Treg expansion in the thoracic cavity two weeks after treatment (**Figure 3D**). A significant expansion of Tr1 cells, however, was still observed in the thoracic cavity and in the spleen (**Figures 3B, C**). As additional control we show that FBZ-treatment of naïve control mice did not modulate Tr1 and Treg populations (white to grey symbols **Figure 3B**: p = 0.55; **Figure 3C**: p = 0.36; **Figure 3D**: p = 0.58; **Figure 3E**: p = 0.48)

In summary these experiments show that vaccination after efficient deworming (**Supplementary Figure S1B**) did not induce a statistically significant elevation of neutralizing anti-HA Ab (**Figure 2B**), did not revert the helminth infection-induced Tr1 cell expansion (**Figures 3B, C**) and did not restore vaccination-induced protection against influenza (**Figure 2C**).

### A Combination of Deworming and Prime-Boost Vaccination With Fluad Restores Influenza Vaccination-Induced Protection in *L. sigmodontis-*Infected Mice

**Figure 4:**
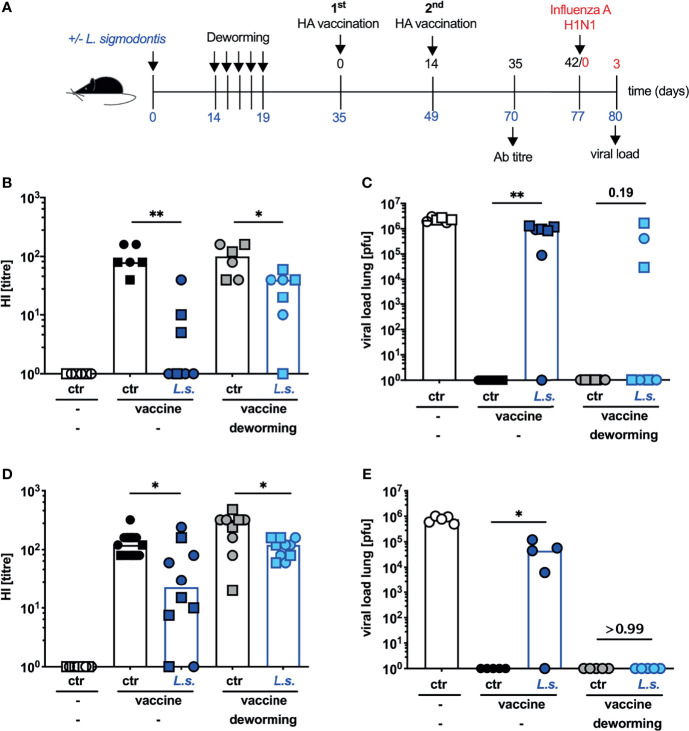
Deworming 2 weeks before prime-boost vaccination with adjuvanted vaccine restores the efficacy of vaccination against influenza. **(A)** Experimental design: C57BL/6 mice were left naive (white, black and grey symbols) or naturally infected with *L. sigmodontis* (dark and light blue symbols). Mice received deworming treatment as a daily s.c. injection with 5 mg FBZ per kg body weight (grey and light blue symbols) for 5 consecutive days on day 14 p.i. – day 19 p.i. or were left untreated. Deworming efficacy is shown in **Supplementary Figure S1**. Mice were i.p. vaccinated with 3.75 µg influenza vaccine either Begripal **(B, C)** or Fluad **(D, E)** on day 35 and day 49 post *L. sigmodontis* infection or were left unvaccinated (white symbols). **(B, D)** HI titres were quantified 21 days after the second vaccination. **(C, E)** All mice were i.n. infected with 1 x 10^3^ PFU 2009 pH1N1 influenza A virus 28 days after the 2^nd^ vaccination. Influenza virus burden in the lungs was quantified at day 3 post challenge infection. Shown are combined results from 2 experiments **(B, C)** or 1 experiment **(D, E)** with n ≥ 3 per group and experiment. Each symbol represents an individual mouse, squares, circles and triangles represent independent experiments, bars show the median and asterisks indicate statistically significant differences applying Mann-Whitney test (*p ≤ 0.05, **p ≤ 0.01, numbers indicate *p*-values). (*L. sigmodontis*, *Litomosoides sigmodontis*; s.c., subcutaneous; FBZ, Flubendazole; p.i., post infection; i.p., intraperitoneal; HI, hemagglutination inhibition; i.n., intra nasal; PFU, plaque forming units).

From the translational point of view the ultimate goal of vaccination is the establishment of sterile immunity to influenza virus infection despite pre-existing helminth infections. In a previous study, we observed that neither introduction of a prime-boost immunization nor application of the MF59-adjuvanted anti-influenza vaccine Fluad restored vaccination efficacy in helminth-infected mice (unpublished observation). As also drug-induced deworming alone did not restore vaccination efficacy, we next asked if a combination of both approaches would outcompete helminth-induced immunomodulation. To this end, mice were naturally infected with *L. sigmodontis*, FBZ-treated and vaccinated 2 weeks after the final treatment. Instead of a single vaccination that is usually applied for influenza vaccination of humans, mice received 2 consecutive injections (**Figure 4A**). This prime-boost vaccination regimen with the non-adjuvanted influenza vaccine Begripal did not elevate the Ab response to vaccination, nor restore the protection in helminth-infected but untreated mice (**Figures 4B, C**) as we observed before (unpublished data). FBZ-treatment of helminth-infected mice led to a partial restoration of the prime-boost vaccination efficacy. Although the HI titre was still significantly lower in FBZ-treated and helminth-infected mice compared to the FBZ-treated non-helminth infected control group, sterile protection against challenge infection with 2009 pH1N1 was established in 57% of the mice (**Figure 4C**). Likewise, a prime-boost vaccination with the MF59-adjuvated influenza vaccine Fluad (25) did not restore the Ab titre to normal levels (**Figure 4D**) nor establish sterile protection from challenge infection with 2009 pH1N1 influenza A virus in helminth-infected mice (**Figure 4E**). However, FBZ-treatment 2 weeks before the prime-boost vaccination with Fluad resulted in sterile immunity in 100% of the formerly helminth-infected mice (**Figure 4E**). Again FBZ-treatment as such did not modulate the vaccination-response of non-helminth infected mice (**Figures 4B, C**, **E**: p > 0.99, **Figure 4D**: p = 0.078).

## Discussion

Using *L. sigmodontis-*infected mice as an established model for chronic human filarial infection (10–12), we tried to restore responsiveness to vaccination against influenza by anthelmintic treatment with FBZ. In a first approach, we vaccinated the mice during the treatment. Simultaneous application of anthelmintic treatment and vaccination is commonly used in agriculture and would also be the most feasible approach in the field. However, this regimen did neither rescue neutralizing Ab responses nor vaccination-induced protection from challenge infections with 2009 pH1N1 influenza A virus. This failure is most likely reflecting the presence of living worms and their ongoing modulatory capacity at the moment of vaccination. In a second approach, we delayed the vaccination until the FBZ-treatment successfully eliminated the parasitic adults in the thoracic cavity. Again, this vaccination did not prevent 2009 pH1N1 influenza A virus replication in the lungs of the treated, previously helminth-infected mice.

Several human studies attempted to restore vaccination responses in helminth-endemic areas by drug-induced deworming yielding heterogenous results. While some studies reported an elevation of vaccination response after treatment (26–28), others observed partial restoration (29) or did not report a clear effect of de-worming (30, 31). One technical problem that is immanent to human studies is the efficacy and sustainability of deworming as well as the correct diagnosis of helminth infections, including intestinal and tissue-dwelling parasites, in the study subjects. Studies reporting that a single dose of albendazole did not elevate vaccination responses to influenza, -meningococcal or -cholera vaccination, showed also that worm burden in untreated and treated cohorts was unchanged at the time point of analysis (30, 31). Although results obtained in murine studies cannot be directly translated to the human situation, the advantage of the mouse system in this context is that efficacy and kinetics of drug-induced helminth elimination can be tested and superinfections with additional helminths or other pathogens are excluded. Moreover, the efficacy of a vaccination against seasonal influenza can be directly tested by challenge infections of vaccinated mice with the human pathogenic 2009 pH1N1 influenza A virus. Although the mouse system allows the control of many parameters, we still observe a high inter- and intra- experimental variation regarding the HI titres in response to vaccination and the influenza virus load in the lung of challenge-infected mice throughout our results presented here as well as in our previous study (21). Despite this system-immanent variation, the outcome of concurrent helminth infection in (i) reducing the Ab response to anti-influenza vaccination and in (ii) abrogating vaccination-induced protection against a 2009 pH1N1 influenza A virus challenge infection is highly reproducible. This outcome was observed in every experiment throughout this study and in our previous study (21).

In the current study, we treated the mice with FBZ, an anthelminthic drug that belongs to the benzimidazole family, interferes with the microtubule formation. FBZ is highly efficient against gastrointestinal nematodes (32–34) and also against filarial infections (35–38). Especially the s.c. application of FBZ was shown to kill *L. sigmodontis* parasitic adults in infected jirds recently (39). Consenting with these findings, we report that FBZ-treatment of *L. sigmodontis-*infected mice resulted in complete eradication of viable parasitic adults 2 weeks after therapy onset. However, the suppressive state was not reverted with the death of the worms, showing that helminth-induced immunomodulation was sustained in the absence of viable parasites.

Several lines of evidence suggest that even after successful anthelmintic treatment of mice a refractory state needs to be passed until the immune system reverted to normal function. *Schistosoma mansoni* infection impaired the T cell response to a DNA-based multi T cell epitope HIV vaccine (40). Praziquantel (PZQ) treatment 4 weeks prior the immunization did not lead to complete restoration of the cellular response, while an 8-week period between treatment and vaccination elevated the HIV peptide-specific T cell response to the levels observed in non-helminth-infected mice (41). Similarly, PZQ treatment 2,5 weeks before vaccination did not rescue the Ab response to an HIV gp140 Env protein that was suppressed in *S. mansoni*-infected mice (42). In a kinetic study, Chen and co-workers showed that prolonging the period between PZQ-treatment of *S. japonicum*-infected mice and application of recombinant hepatitis B virus (HBV) vaccine gradually rescued the HB-specific Ab responses and HB-specific cytokine production by splenocytes (43).

In line with these findings, we reported sustained immunomodulation after immune-driven termination of an *L. sigmodontis* infection in mice. The Ab response to model antigen immunization was suppressed in fully susceptible BALB/c mice that were immunized 4 months after the disappearance of *L. sigmodontis* microfilaria from the peripheral circulation (17). Semi-susceptible C57BL/6 mice do not develop microfilariae and start to clear the infection from day 35 onwards (10, 13, 14). Still, the Ab response to a vaccination against influenza that was applied 100 days post *L. sigmodontis* infection, i.e., in the absence of viable parasites, was suppressed (21). This sustained suppressive state in *L. sigmodontis*-infected mice was correlated to an expansion of both Foxp3^+^ Treg and CD4^+^CD49b^+^LAG3^+^ Tr1 cells. Thereby Treg expanded only locally, in the thoracic cavity at the site of infection. Transient Treg depletion using the Depletion of Regulatory T cell (DEREG) mouse model (44), either during vaccination or during initial helminth-infection did not restore Ab response to model antigen immunization (17) or to vaccination against influenza (21). By contrast, IL-10-producing CD4^+^CD49b^+^LAG3^+^ Tr1 cells expanded both in the thoracic cavity and systemically in the spleen. Systemic expansion of Tr1 cells was apparent 90 days post infection and blockade of the Tr1 cell key cytokine IL-10 during the vaccination partially restored the Ab response to model antigens (16) and to vaccination against influenza (21) also restoring vaccination-induced protection from a challenge infection with 2009 pH1N1 influenza A virus.

In line with these previous findings, we observed a sustained expansion of Tr1 cells in the spleens of *L. sigmodontis-*infected mice in the current study, that was apparent 2 weeks after FBZ-treatment, i.e., in the absence of living worms. Interestingly, the same FBZ-treatment resulted in the contraction of the Foxp3^+^ Treg compartment, strongly suggesting that Treg expansion depended on the presence of viable helminth parasites while the Tr1 cell population remained expanded either after immune-driven or drug-induced termination of helminth-infection. We suggest that expanding Tr1 cells contribute to the suppressed vaccination responses that we observed after efficient clearance of the worm infection. Thereby their continuous presence provides one mechanistic explanation for the “refractory” period between efficient clearance of helminth-infections and the delayed restoration of responsiveness to vaccination observed also by other groups (41–43). However, it should be noted that helminth-induced suppression is most likely caused by a plethora of regulatory pathways with Tr1 cells being one factor among many such as regulatory B cells (45–47), regulatory “checkpoint” receptors (48, 49), myeloid-derived suppressor cells (50) and even microRNAs (51) that have been shown to downmodulate the immune system during helminth infections.

We did not test if Tr1 cell frequencies would eventually revert to naïve levels at later time points after drug-induced deworming and if that would correlate with the restoration of vaccination efficacy in the FBZ-treated mice because the aim of the current study was to perform prospective studies to evaluate potential vaccination strategies that would function in helminth-endemic areas. We did not study longer periods between deworming and vaccination because the risk of re-infection during a period longer than 2 weeks between treatment and vaccination renders that approach impracticable. One strategy to outcompete the suppressive state inflicted by concurrent helminth-infection is the application of improved vaccination regimen. In this context, life carrier vaccines such as an antigen-expressing live *Salmonella* strains or irradiated sporozoites conferred protection against a *P. berghei* or *P. chabaudi* sporozoite challenge infection in helminth-infected mice in settings where DNA- or protein-based vaccines failed (15, 52). Likewise, the HIV-specific cellular response to an attenuated HIV-gag expressing *Listeria monocytogenes* vaccine vector was not compromised in helminth-infected mice although protection could not be tested in this study (53).

We have previously tried to improve the influenza vaccination efficacy employing influenza vaccines that are licensed for humans. We observed that both, a prime-boost vaccination with the non-adjuvanted vaccine Begripal or a single vaccination with the MF59-adjuvanted vaccine Fluad elevated the Ab response to vaccination in comparison to a single vaccination with Begripal (unpublished observation). This elevation was observed in uninfected and helminth-infected mice. Interestingly, the elevated Ab response was sufficient to prevent the transient weight loss induced by a challenge infection with influenza A virus that can be used as a readout of morbidity (54). However, it was not sufficient to prevent influenza virus replication in the lung, i.e., sterile immunity was not achieved. In the current study we chose to monitor viral load in the lungs as the most stringent indicator of vaccine efficacy. In line with our previous findings, we observed that neither prime-boost nor adjuvanted anti-influenza vaccination induced sterile immunity to a challenge infection with 2009 pH1N1 influenza A virus in helminth-infected mice. However, mice that were dewormed 14 days prior vaccination and thus helminth-free at the moment of vaccination were protected by a prime-boost vaccination with Fluad.

It should be pointed out again that, as our study was performed in mice, the results cannot be directly translated to the human situation. We rather suggest that our findings establish a “proof of principle” that a combination of both, drug-induced deworming and improved vaccination regimen may provide a first promising strategy to re-establish vaccination-induced protection on the background of concurrent helminth infection. Certainly, this strategy needs to be further investigated in human studies, using anthelmintic treatments that are licensed for humans.

## Concluding Remarks

Our combined results suggest that pre-existing helminth-infections impair the efficacy of vaccinations and especially compromise vaccine-induced protection in mice. Even efficient deworming may not restore responsiveness to vaccination since the suppressive state is preserved in the absence of living parasites. A combination of anthelminthic treatment and improved vaccinations, however, may outcompete helminth-induced immunomodulation.

## Data Availability Statement

The original contributions presented in the study are included in the article/**Supplementary Material**. Further inquiries can be directed to the corresponding author.

## Ethics Statement

The animal study was reviewed and approved by Behörde für Gesundheit und Verbraucherschutz, Hamburg, approval numbers 84/15, 103/2018.

## Author Contributions

MB, GG, NS, and WH conceived and designed the experiments. NS, WH, M-LB, and SS-B performed the experiments. NS, MB, WH, and GG analyzed the data. SS-B and GG contributed reagents/materials/analysis tools. MB, WH, and GG wrote the manuscript. All authors contributed to the article and approved the submitted version.

## Funding

This project was funded by the German Research Association (DFG) grant BR 3754/2-2. NS was funded by the Landesforschungsförderung Hamburg LVV-FV36. The funders had no role in the study design, data analysis and interpretation of the results.

## Conflict of Interest

The authors declare that the research was conducted in the absence of any commercial or financial relationships that could be construed as a potential conflict of interest.

## Publisher’s Note

All claims expressed in this article are solely those of the authors and do not necessarily represent those of their affiliated organizations, or those of the publisher, the editors and the reviewers. Any product that may be evaluated in this article, or claim that may be made by its manufacturer, is not guaranteed or endorsed by the publisher.
