# Supplementary material for: A Combination of Deworming and Prime-Boost Vaccination Regimen Restores Efficacy of Vaccination Against Influenza in Helminth-Infected Mice
Source: Front Immunol. 2021 Dec 21;12:784141. doi: 10.3389/fimmu.2021.784141 (PMC8724120; doi:10.3389/fimmu.2021.784141)
Supplement: Supplementary file 1 [file DataSheet_1.pdf]

Supplementary Figures

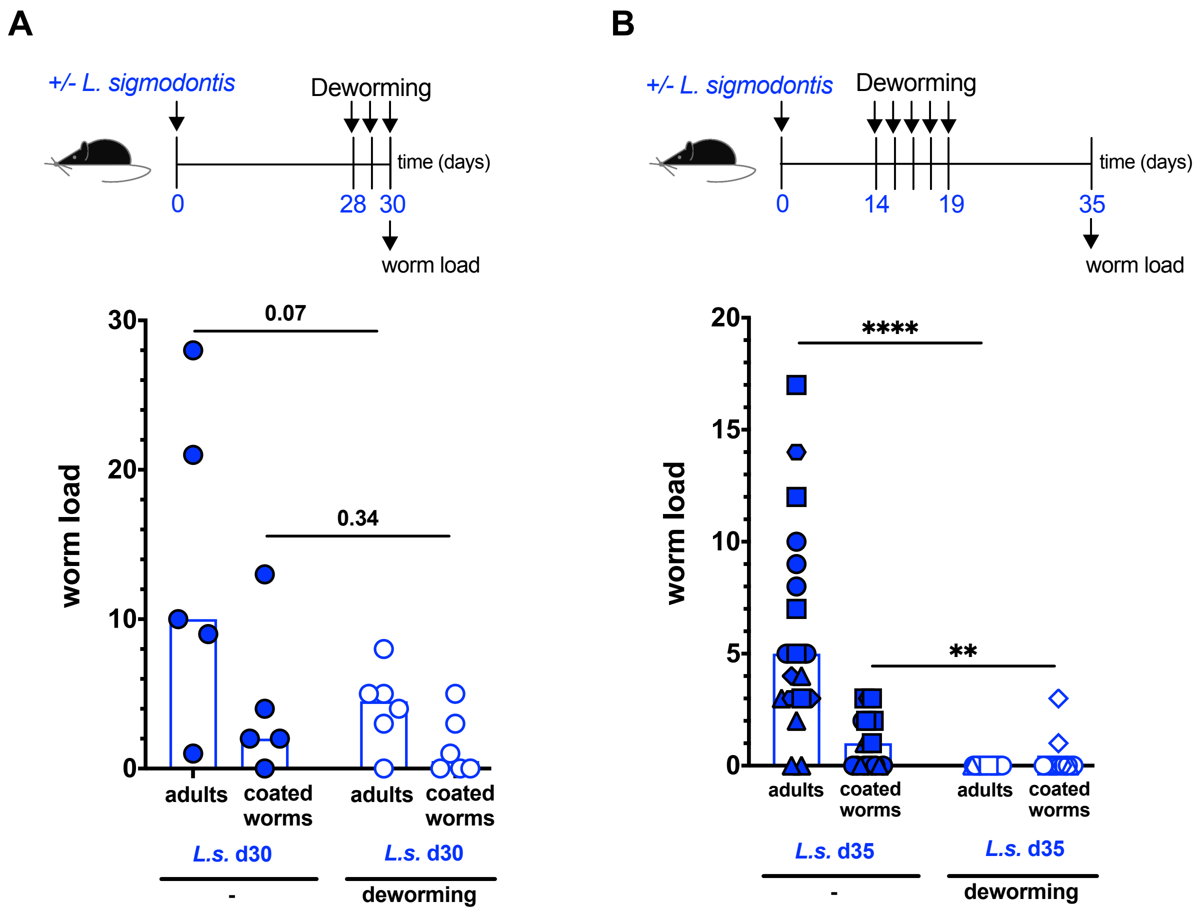

Supplementary Figure S1 related to Figures 1-4: Efficiency of FBZ treatment

C57BL/6 mice were naturally infected with *L. sigmodontis*. **(A)** Mice received 3 consecutive FBZ injections from day 28 p.i. until day 30 p.i. (open symbols) or were left untreated (blue symbols) and were sacrificed the same day to count the worm load. **(B)** Mice received 5 consecutive FBZ injections from day 14 p.i. until day 19 p.i. (open symbols) or were left untreated (blue symbols) and were sacrificed at day 35 p.i. to count the worm load. Shown are results from one experiment with n ≥ 5 per group **(A)** or combined results from 5 independent experiments with n ≥ 2 per group and experiment. **(B)** Each symbol represents an individual mouse, squares, circles, triangles, diamonds and hexagons represent independent experiments, the bars show the median. Asterisks indicate statistically significant differences applying Mann-Whitney test (** $p \leq 0.01$, **** $p \leq 0.0001$) and numbers indicate *p*-values. (Abbreviations: *L. sigmodontis* = *Litomosoides sigmodontis*, FBZ = Flubendazole, p.i. = post infection)

Supplementary Figure S2 related to Figure 3: Gating strategy for Tr1 cells and Treg

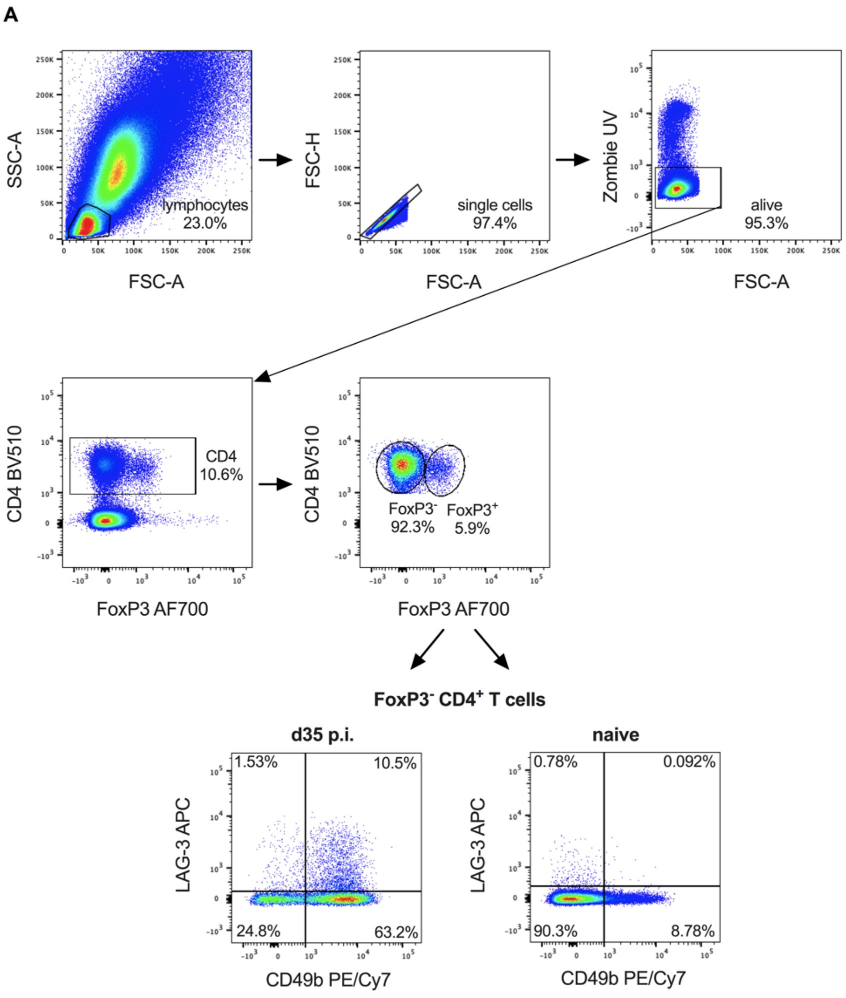

Thoracic cavity cells **(A)** or spleen cells **(B)** were harvested from day 35 *L. sigmodontis* infected or naïve mice that were either dewormed with FBZ or left untreated as indicated in Figure 3. Single cell preparations were stained with Zombie UV to exclude dead cells and with CD4-BV510, LAG-3-APC, CD49b-PE/Cy7 and Foxp3-AF700. Representative dot blots showing the gating strategy. Living $CD4^+$ single cells were further distinguished by their expression of Foxp3. The expression of LAG-3 and CD49b is shown for thoracic cavity cells from naïve and day 35 infected mice. Cells were measured on a LSRII and further analyzed by Flow Jo. (Abbreviations: *L. sigmodontis* = *Litomosoides sigmodontis*, FBZ = Flubendazole, UV = ultra violet, CD = cluster of differentiation, BV = Brilliant Violet, LAG-3 = lymphocyte activation gene-3, APC = Allophycocyanain, PE = Phycoerythrin, AF = Alexa Fluor, Tr1 = type 1 regulatory T cells, Treg = regulatory T cells, Foxp3 = Forkhead-Box-Protein P3)
