# Supplementary material for: A Combination of Deworming and Prime-Boost Vaccination Regimen Restores Efficacy of Vaccination Against Influenza in Helminth-Infected Mice
Source: Front Immunol. 2021 Dec 21;12:784141. doi: 10.3389/fimmu.2021.784141 (PMC8724120; doi:10.3389/fimmu.2021.784141)
Supplement: Supplementary file 2 [file DataSheet_2.pdf]

Supplementary Material: Dot Plots of every measure point shown in Figure 3.

Thoracic cavity cells or spleen cells were harvested from day 35 *L. sigmodontis* infected or naïve mice that were either dewormed with FBZ or left untreated Single cell preparations were stained with Zombie UV to exclude dead cells and with CD4-BV510, LAG-3-APC, CD49b-PE/Cy7 and Foxp3-AF700. Representative dot blots showing the gating strategy are provided as supplementary Figure S2. Living $CD4^{+}$ single cells were further distinguished by their expression of Foxp3. The expression of LAG-3 and CD49b is shown for thoracic cavity cells from naïve and day 35 infected mice. Cells were measured on a LSRII and further analyzed by Flow Jo. Example Dot Plots for quantification of Treg as $CD4^{+}FoxP3^{+}$ cells and Tr1 cells as $CD4^{+}Foxp3^{-}$ and additionally $LAG3^{+}CD49b^{+}$ cells were provided in supplementary Figure S2.

Here every Dot Plot of every measurement shown in Figure 3 is provided.

Thereby

Non-infected mice are indicated as naïve

Flubendazole-treated naïve mice are indicated as FBZ or naïve + FBZ

Litomosoides sigmodontis- infected mice are indicated as Lito

Litomosoides sigmodontis- infected mice and FBZ-treated mice are indicated as Lito/FBZ or Lito + FBZ

Dot Plots used for Figure 3B (Tr1 cells in the thoracic cavity):

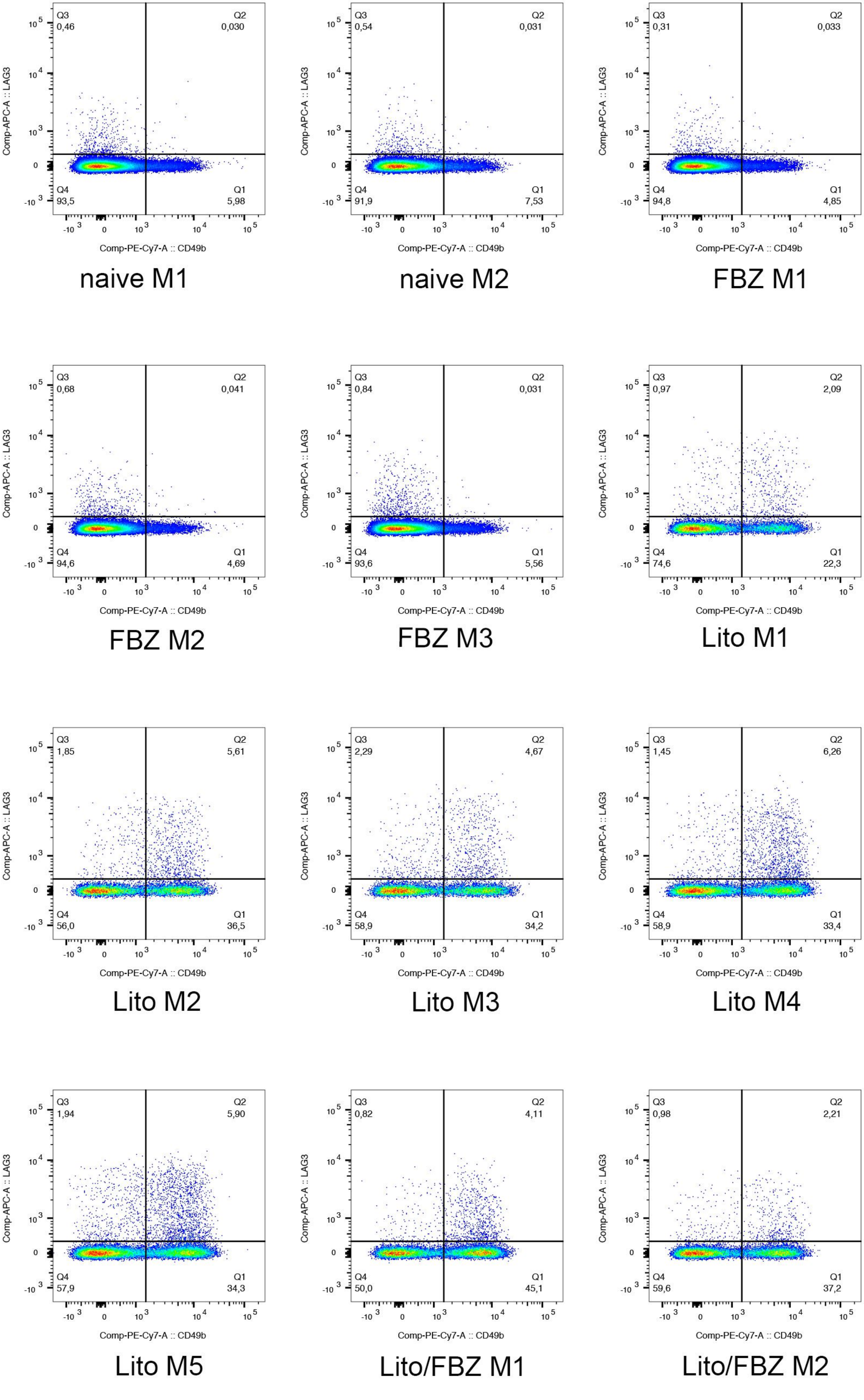

Comp-APC-A :: LAG3
Comp-PE-Cy7-A :: CD49b
naive M1
Q3 0,46
Q2 0,030
Q4 93,5
Q1 5,98
naive M2
Q3 0,54
Q2 0,031
Q4 91,9
Q1 7,53
FBZ M1
Q3 0,31
Q2 0,033
Q4 94,8
Q1 4,85
FBZ M2
Q3 0,68
Q2 0,041
Q4 94,6
Q1 4,69
FBZ M3
Q3 0,84
Q2 0,031
Q4 93,6
Q1 5,56
Lito M1
Q3 0,97
Q2 2,09
Q4 74,6
Q1 22,3
Lito M2
Q3 1,85
Q2 5,61
Q4 56,0
Q1 36,5
Lito M3
Q3 2,29
Q2 4,67
Q4 58,9
Q1 34,2
Lito M4
Q3 1,45
Q2 6,26
Q4 58,9
Q1 33,4
Lito M5
Q3 1,94
Q2 5,90
Q4 57,9
Q1 34,3
Lito/FBZ M1
Q3 0,82
Q2 4,11
Q4 50,0
Q1 45,1
Lito/FBZ M2
Q3 0,98
Q2 2,21
Q4 59,6
Q1 37,2

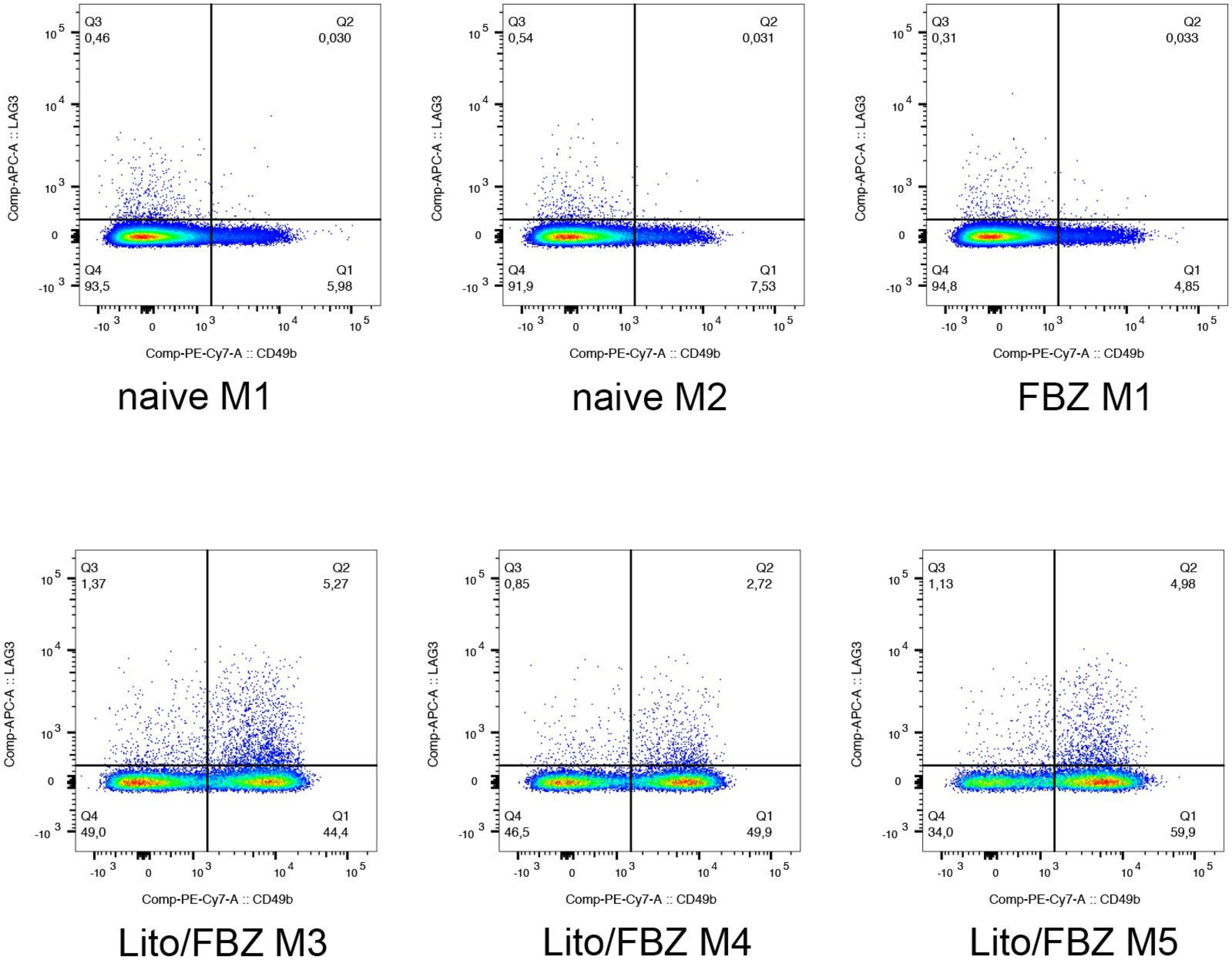

Q3 0,46
Q2 0,030
Q4 93,5
Q1 5,98
naive M1
Q3 0,54
Q2 0,031
Q4 91,9
Q1 7,53
naive M2
Q3 0,31
Q2 0,033
Q4 94,8
Q1 4,85
FBZ M1
Q3 1,37
Q2 5,27
Q4 49,0
Q1 44,4
Lito/FBZ M3
Q3 0,85
Q2 2,72
Q4 46,5
Q1 49,9
Lito/FBZ M4
Q3 1,13
Q2 4,98
Q4 34,0
Q1 59,9
Lito/FBZ M5
Comp-APC-A :: LAG3
Comp-PE-Cy7-A :: CD49b

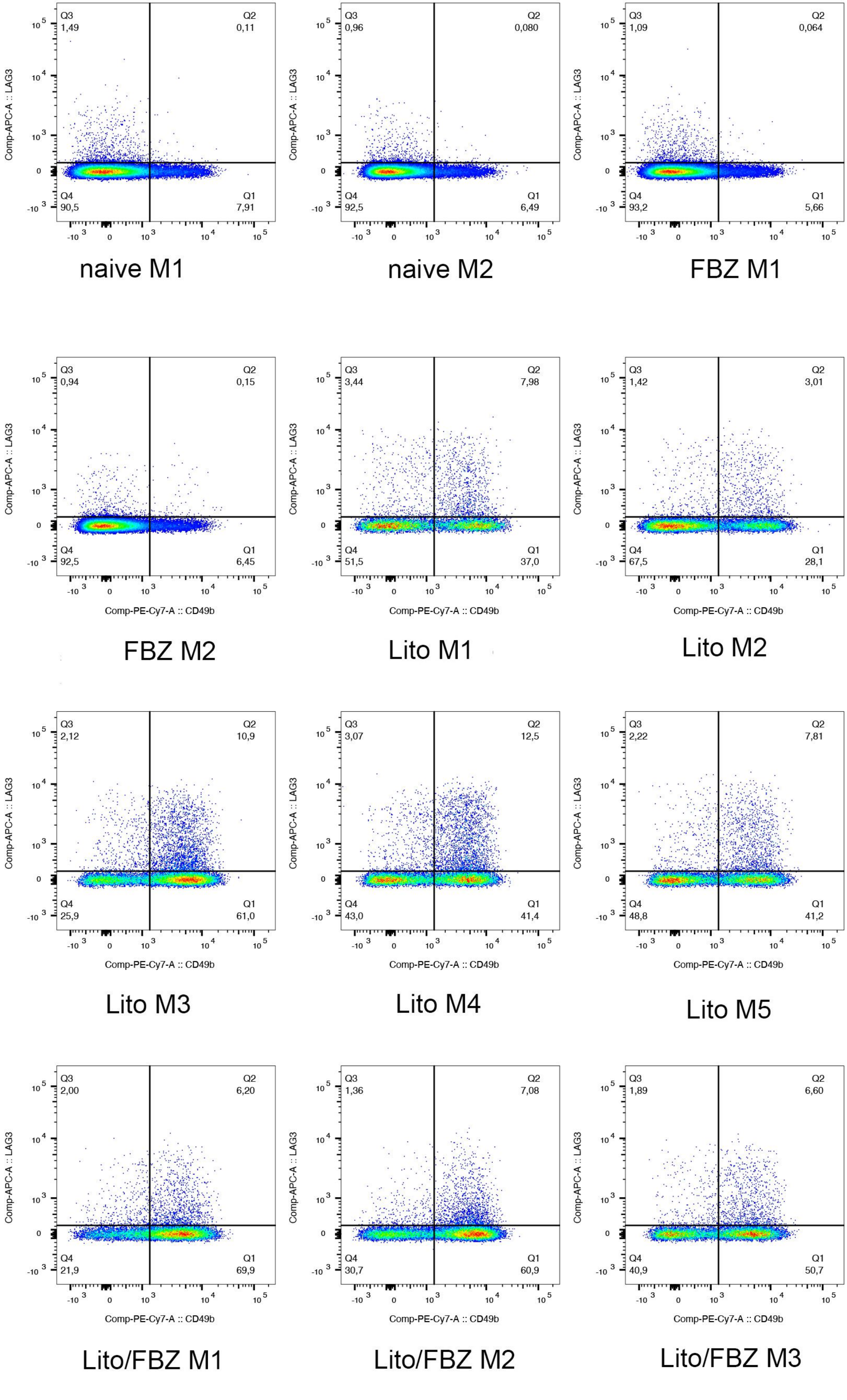
Comp-APC-A :: LAG3
Comp-PE-Cy7-A :: CD49b
Q3 1,49 Q2 0,11 Q4 90,5 Q1 7,91
naive M1
Q3 0,96 Q2 0,080 Q4 92,5 Q1 6,49
naive M2
Q3 1,09 Q2 0,064 Q4 93,2 Q1 5,66
FBZ M1
Q3 0,94 Q2 0,15 Q4 92,5 Q1 6,45
FBZ M2
Q3 3,44 Q2 7,98 Q4 51,5 Q1 37,0
Lito M1
Q3 1,42 Q2 3,01 Q4 67,5 Q1 28,1
Lito M2
Q3 2,12 Q2 10,9 Q4 25,9 Q1 61,0
Lito M3
Q3 3,07 Q2 12,5 Q4 43,0 Q1 41,4
Lito M4
Q3 2,22 Q2 7,81 Q4 48,8 Q1 41,2
Lito M5
Q3 2,00 Q2 6,20 Q4 21,9 Q1 69,9
Lito/FBZ M1
Q3 1,36 Q2 7,08 Q4 30,7 Q1 60,9
Lito/FBZ M2
Q3 1,89 Q2 6,60 Q4 40,9 Q1 50,7
Lito/FBZ M3

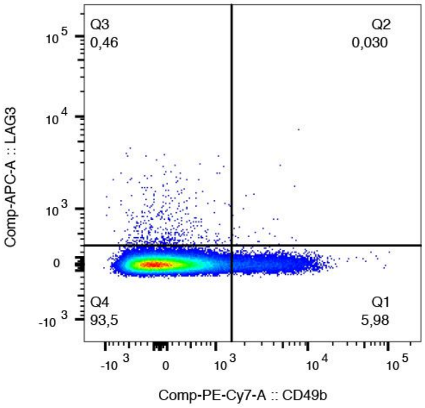

naive M1

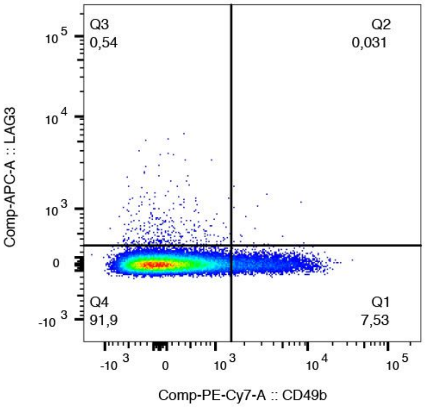

naive M2

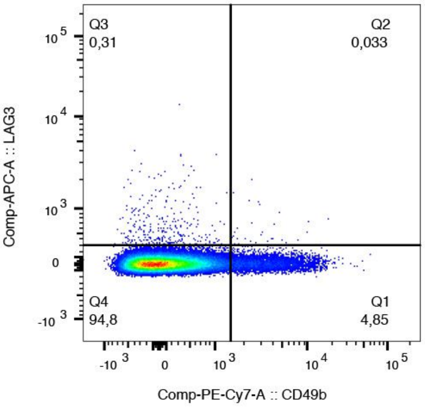

FBZ M1

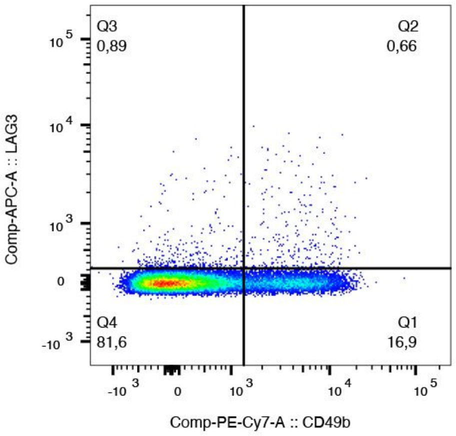

Lito/FBZ M4

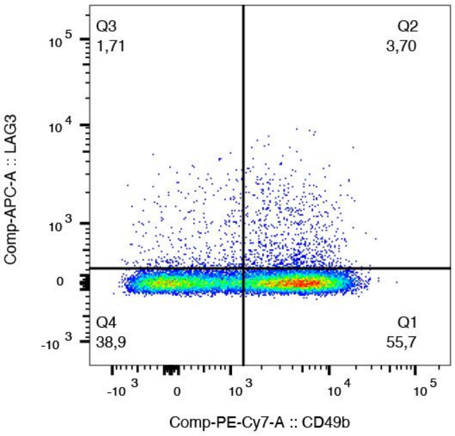

Lito/FBZ M5

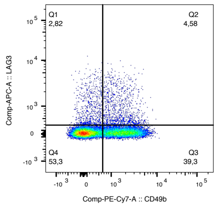

TC_Lito 1.fcs
non FoxP3
22868

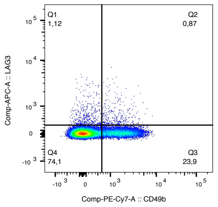

TC_Lito 2.fcs
non FoxP3
35260

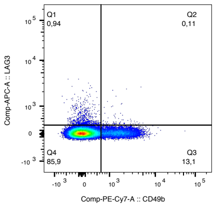

TC_Lito 3.fcs
non FoxP3
69980

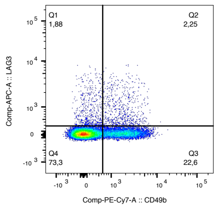

TC_Lito 4.fcs
non FoxP3
28338

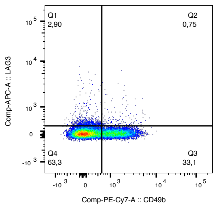

TC_Lito 5.fcs
non FoxP3
23445

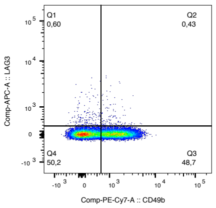

TC_Lito+FBZ 1.fcs
non FoxP3
26961

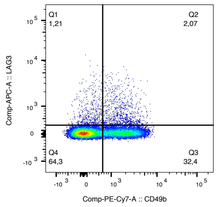

TC_Lito+FBZ 2.fcs
non FoxP3
40338

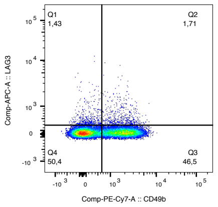

TC_Lito+FBZ 3.fcs
non FoxP3
30845

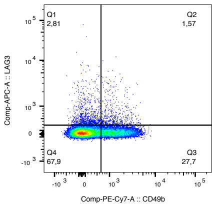

TC_Lito+FBZ 4.fcs
non FoxP3
32998

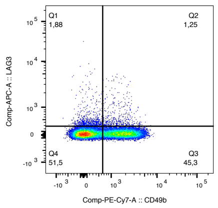

TC_Lito+FBZ 5.fcs
non FoxP3
32016

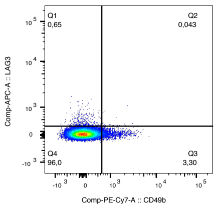

TC_Naiv 1+2.fcs
non FoxP3
42297

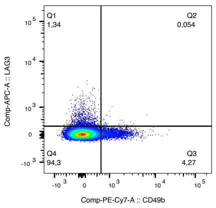

TC_naiv 3.fcs
non FoxP3
70095

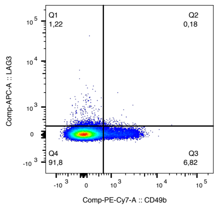

TC_naiv 4+5.fcs
non FoxP3
71458

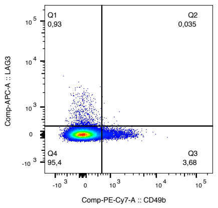

TC_naiv+FBZ 1.fcs
non FoxP3
68942

Dot Plots used for Figure 3C (Tr1 cells in the spleen):

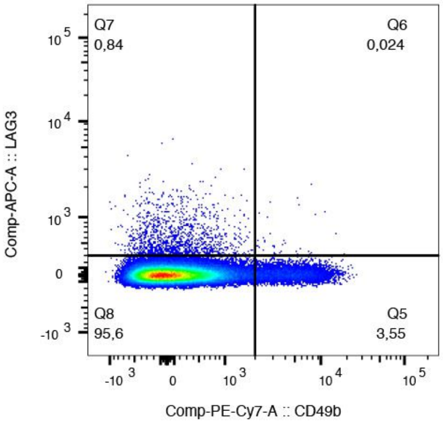

naive M1

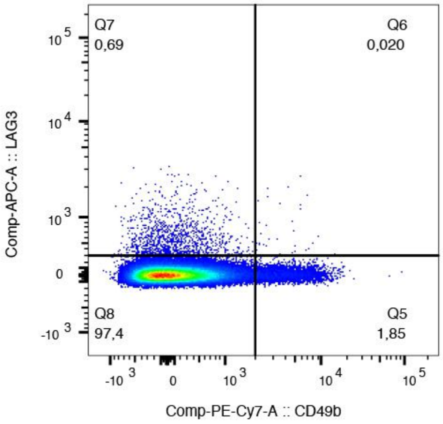

naive M2

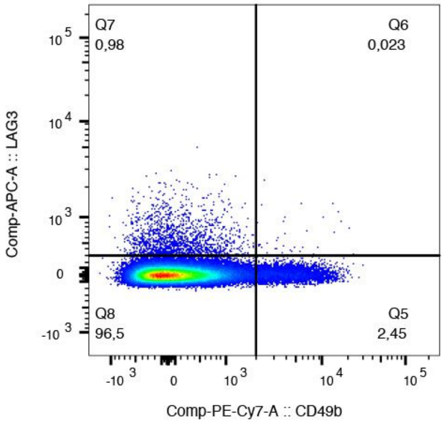

naive M3

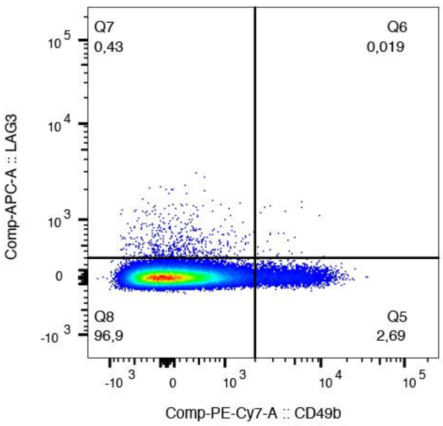

naive M4

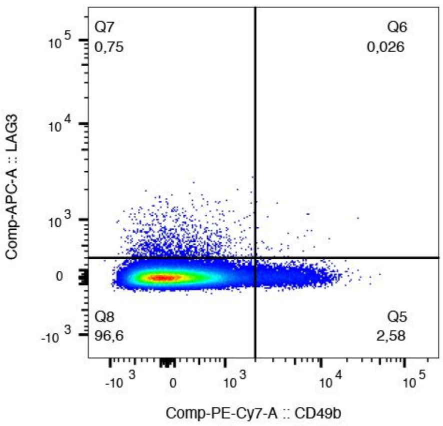

naive M5

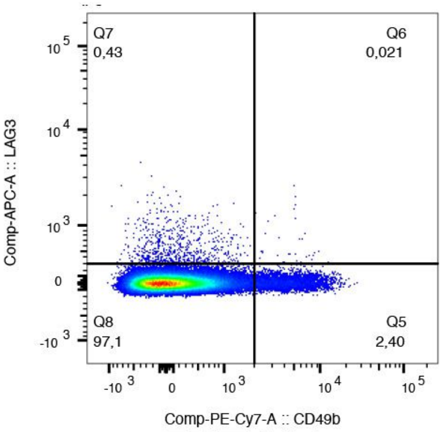

FBZ M1

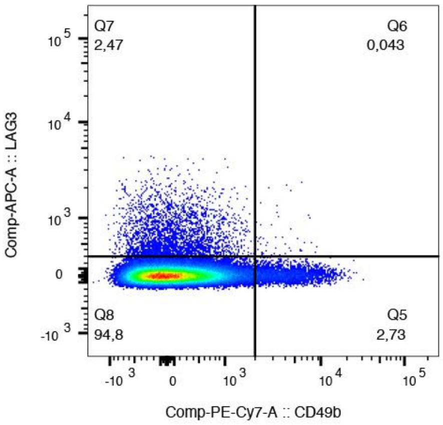

FBZ M2

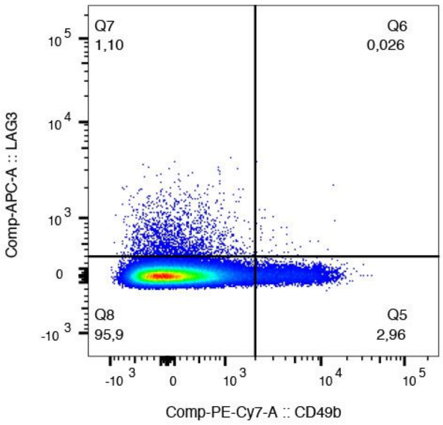

FBZ M3

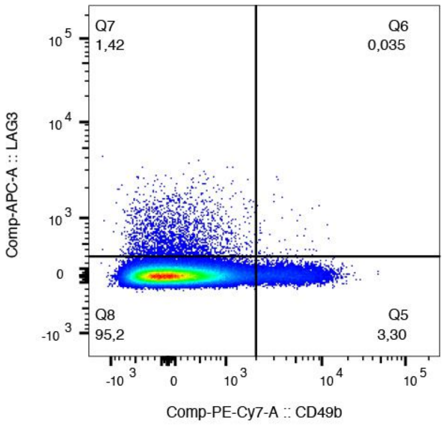

FBZ M4

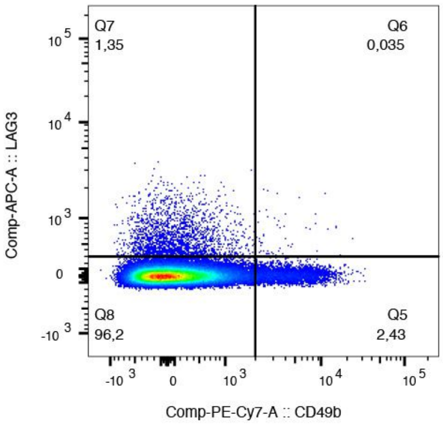

FBZ M5

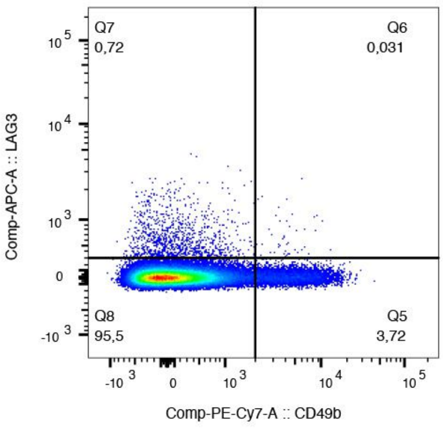

Lito M1

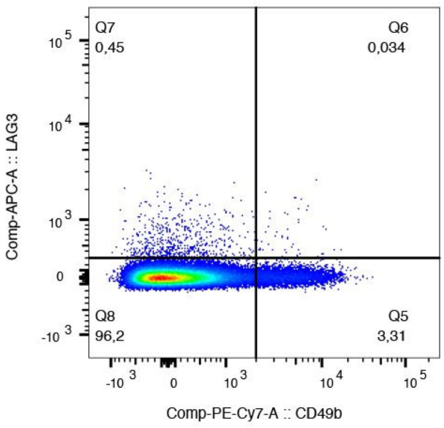

Lito M2

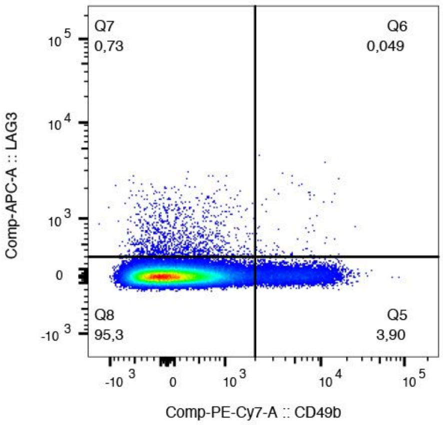

Lito M3

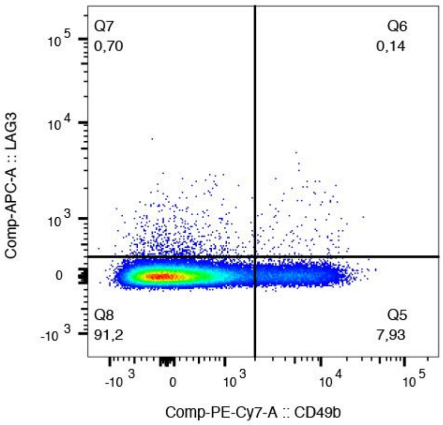

Lito M4

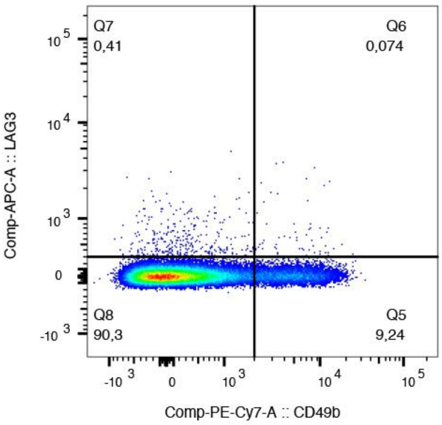

Lito M5

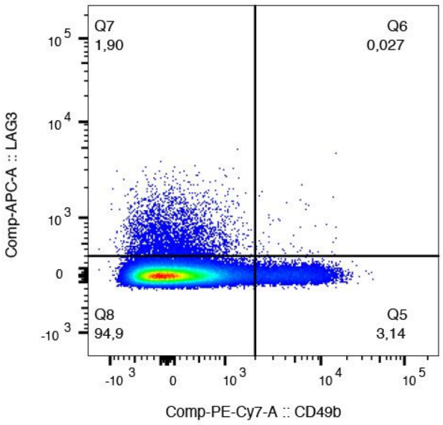

Lito/FBZ M1

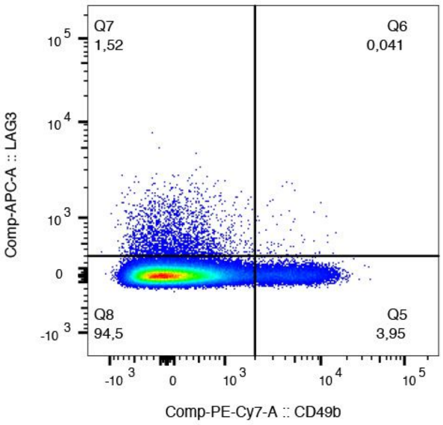

Lito/FBZ M2

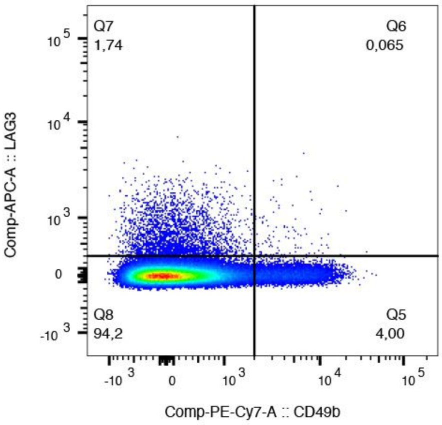

Lito/FBZ M3

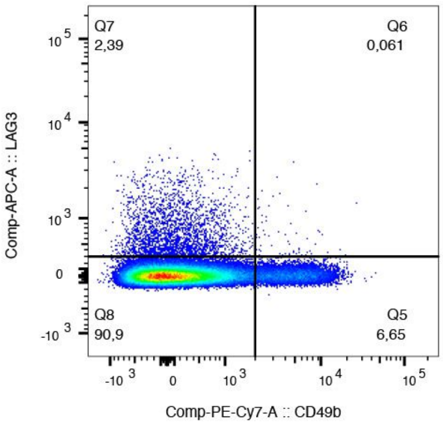

Lito/FBZ M4

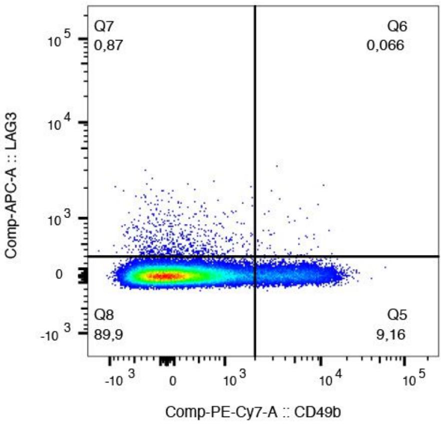

Lito/FBZ M5

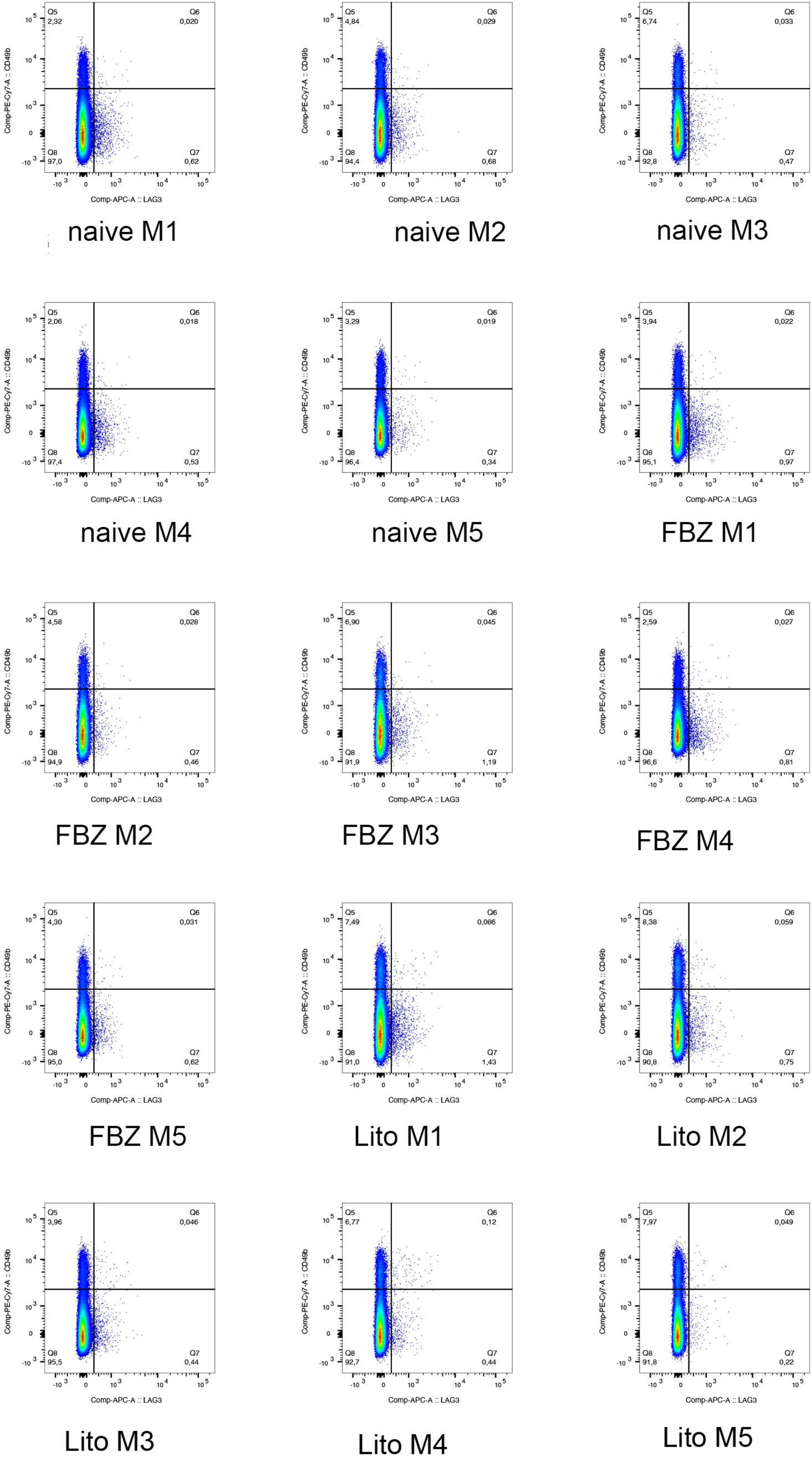
Comp-PE-Cy7-A :: CD49b
Comp-APC-A :: LAG3
Q5 2,32 Q6 0,020 Q8 97,0 Q7 0,62
naive M1
Q5 4,84 Q6 0,029 Q8 94,4 Q7 0,68
naive M2
Q5 6,74 Q6 0,033 Q8 92,8 Q7 0,47
naive M3
Q5 2,06 Q6 0,018 Q8 97,4 Q7 0,53
naive M4
Q5 3,29 Q6 0,019 Q8 96,4 Q7 0,34
naive M5
Q5 3,94 Q6 0,022 Q8 95,1 Q7 0,97
FBZ M1
Q5 4,58 Q6 0,028 Q8 94,9 Q7 0,46
FBZ M2
Q5 6,90 Q6 0,045 Q8 91,9 Q7 1,19
FBZ M3
Q5 2,59 Q6 0,027 Q8 96,6 Q7 0,81
FBZ M4
Q5 4,30 Q6 0,031 Q8 95,0 Q7 0,62
FBZ M5
Q5 7,49 Q6 0,086 Q8 91,0 Q7 1,43
Lito M1
Q5 8,38 Q6 0,059 Q8 90,8 Q7 0,75
Lito M2
Q5 3,96 Q6 0,046 Q8 95,5 Q7 0,44
Lito M3
Q5 6,77 Q6 0,12 Q8 92,7 Q7 0,44
Lito M4
Q5 7,97 Q6 0,049 Q8 91,8 Q7 0,22
Lito M5

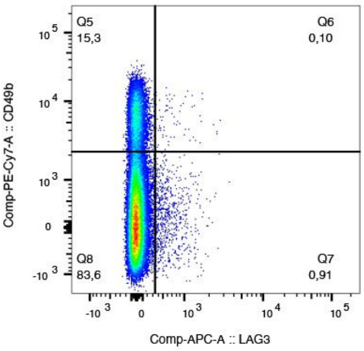

Lito/FBZ M1

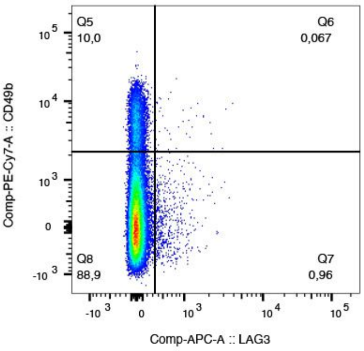

Lito/FBZ M2

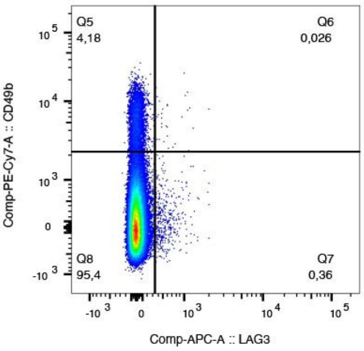

Lito/FBZ M3

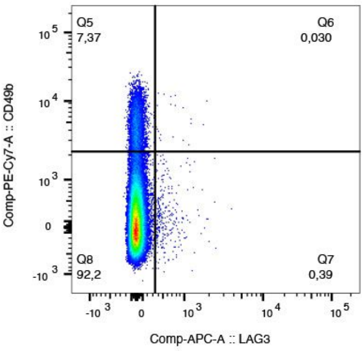

Lito/FBZ M4

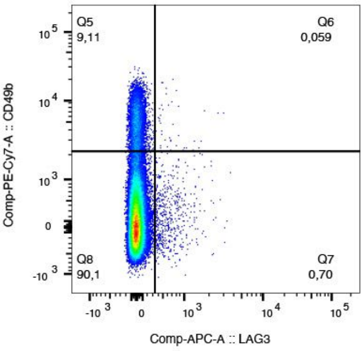

Lito/FBZ M5

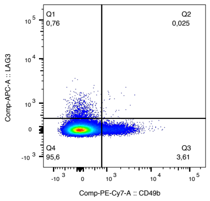

Spleen_Lito 2.fcs
non FoxP3
130171

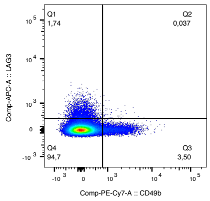

Spleen_Lito 3.fcs
non FoxP3
134033

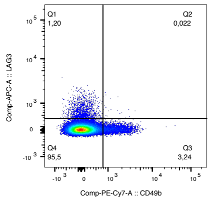

Spleen_Lito 4.fcs
non FoxP3
106779

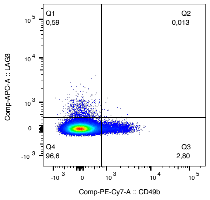

Spleen_Lito 5.fcs
non FoxP3
135553

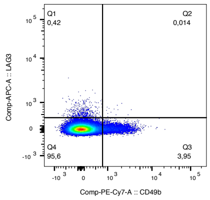

Spleen_Lito+FBZ 1.fcs
non FoxP3
116033

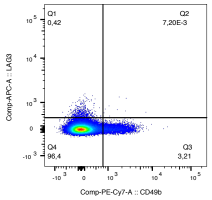

Spleen_Lito+FBZ 2.fcs
non FoxP3
138849

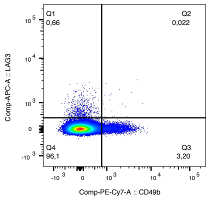

Spleen_Lito+FBZ 3.fcs
non FoxP3
91512

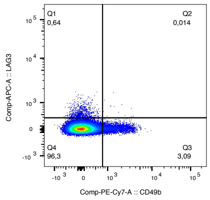

Spleen_Lito+FBZ 4.fcs
non FoxP3
111111

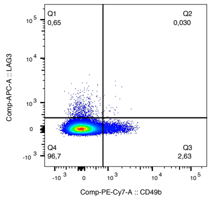

Spleen_Lito+FBZ 5.fcs
non FoxP3
89361

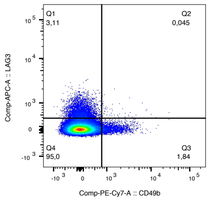

Spleen_Naiv 1.fcs
non FoxP3
123259

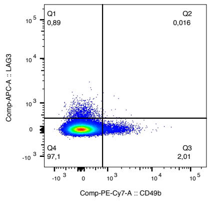

Spleen_Naiv 2.fcs
non FoxP3
116847

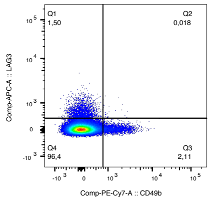

Spleen_Naiv 3.fcs
non FoxP3
142258

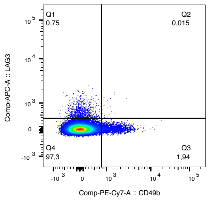

Spleen_Naiv 4.fcs
non FoxP3
118702

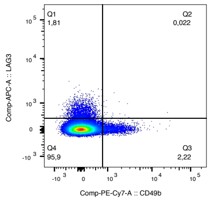

Spleen_Naiv+FBZ 1.fcs
non FoxP3
113394

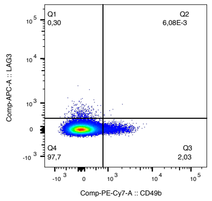

Spleen_Naiv+FBZ 2.fcs
non FoxP3
115062

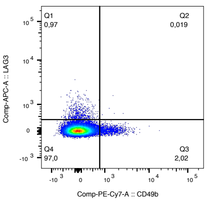

Spleen_Naiv+FBZ 3.fcs
non FoxP3
58460

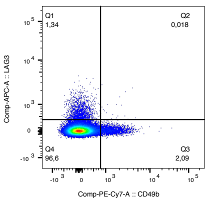

Spleen_Naiv+FBZ 4.fcs
non FoxP3
104741

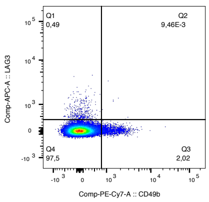

Spleen_Naiv+FBZ 5.fcs
non FoxP3
74012

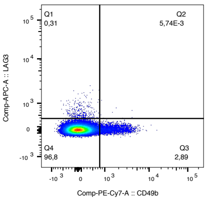

Spleen_naiv 5.fcs
non FoxP3
104522

Dot Plots used for Figure 3D (Treg in the thoracic cavity):

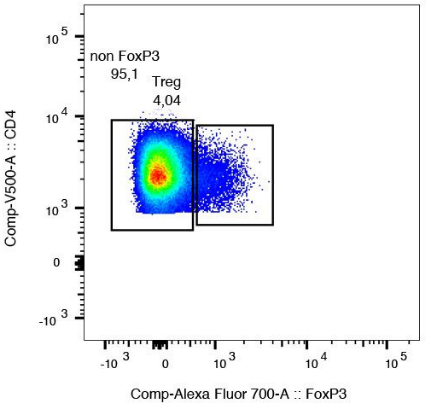

naive M1

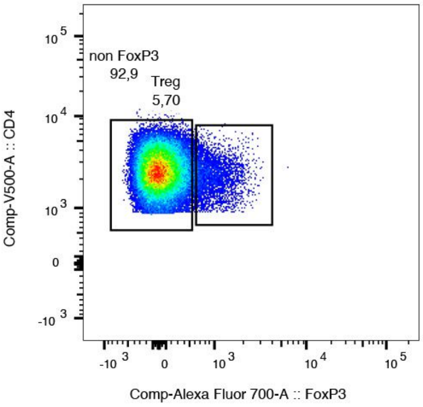

naive M2

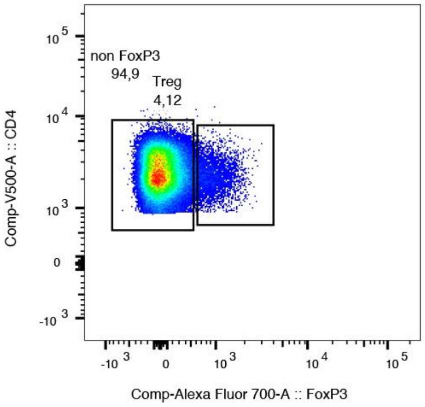

FBZ M1

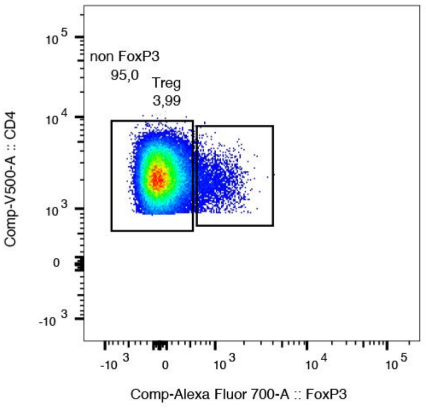

FBZ M2

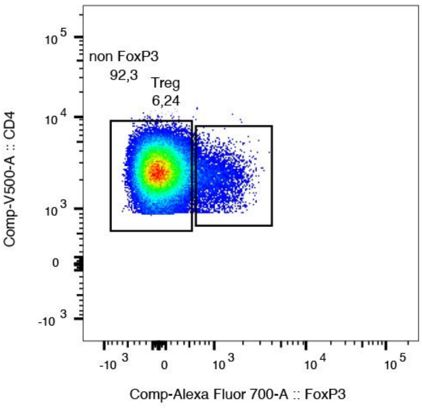

FBZ M3

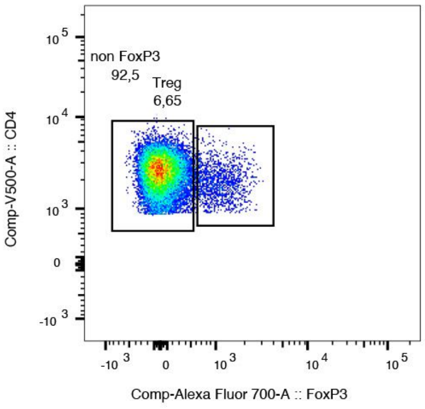

Lito M1

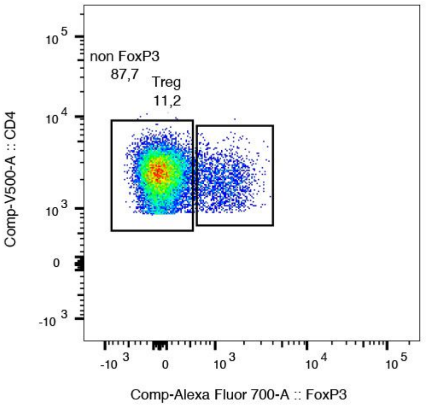

Lito M2

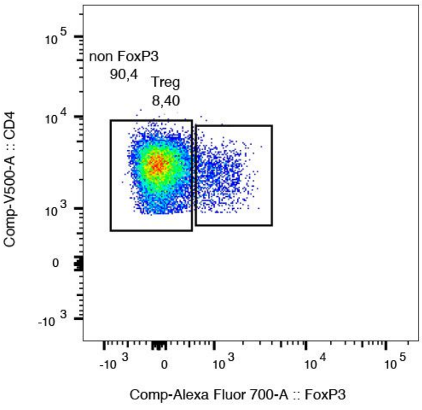

Lito M3

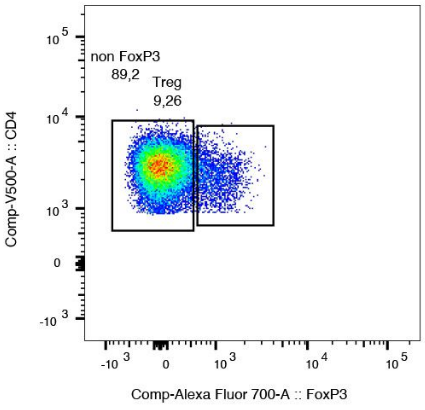

Lito M4

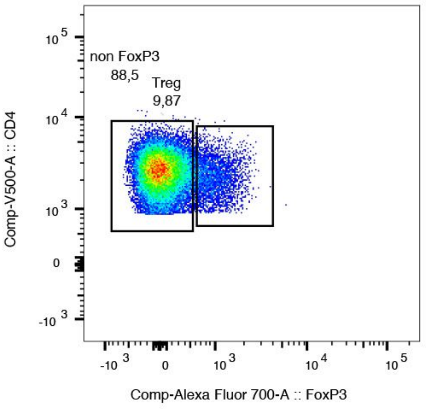

Lito M5

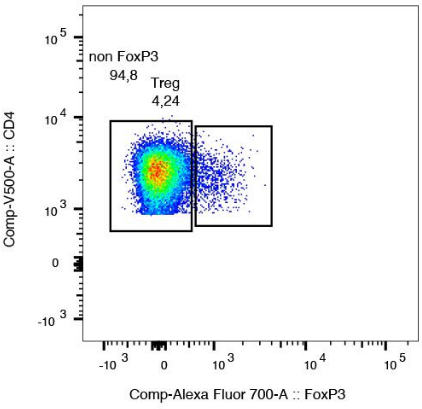

Lito/FBZ M1

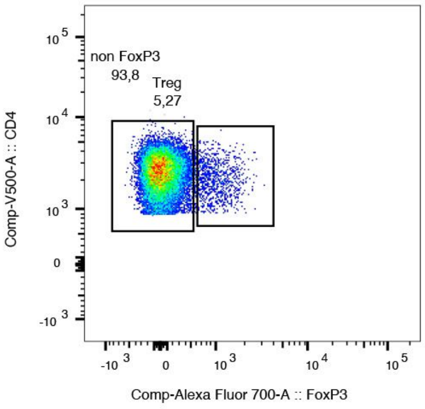

Lito/FBZ M2

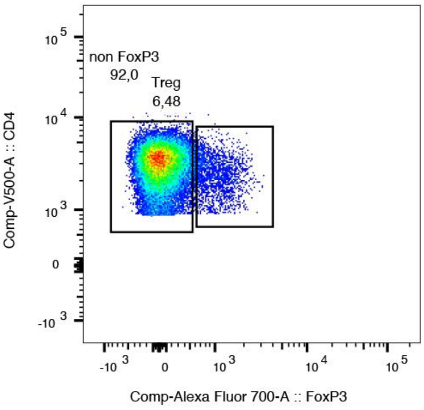

Lito/FBZ M3

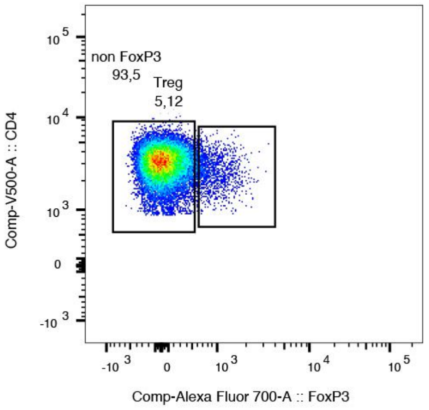

Lito/FBZ M4

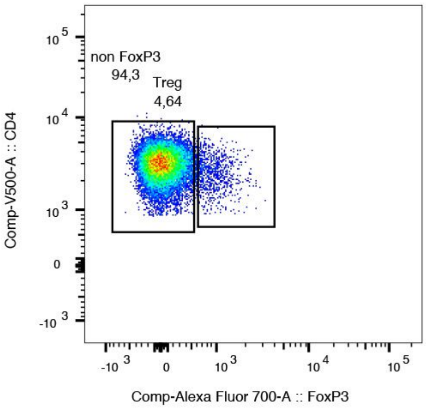

Lito/FBZ M5

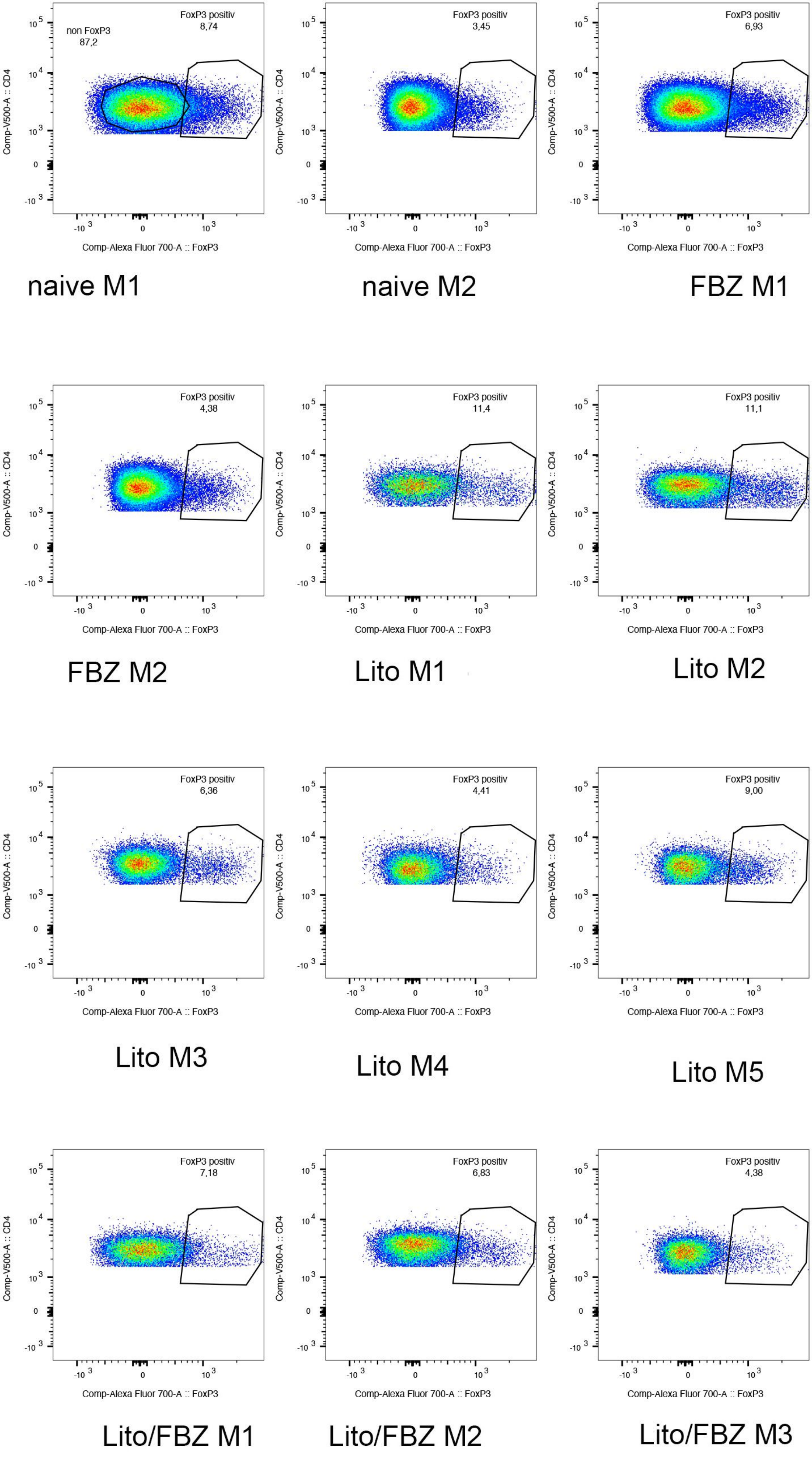

non FoxP3
87,2
FoxP3 positiv
8,74
naive M1
FoxP3 positiv
3,45
naive M2
FoxP3 positiv
6,93
FBZ M1
FoxP3 positiv
4,38
FBZ M2
FoxP3 positiv
11,4
Lito M1
FoxP3 positiv
11,1
Lito M2
FoxP3 positiv
6,36
Lito M3
FoxP3 positiv
4,41
Lito M4
FoxP3 positiv
9,00
Lito M5
FoxP3 positiv
7,18
Lito/FBZ M1
FoxP3 positiv
6,83
Lito/FBZ M2
FoxP3 positiv
4,38
Lito/FBZ M3
Comp-V500-A :: CD4
Comp-Alexa Fluor 700-A :: FoxP3

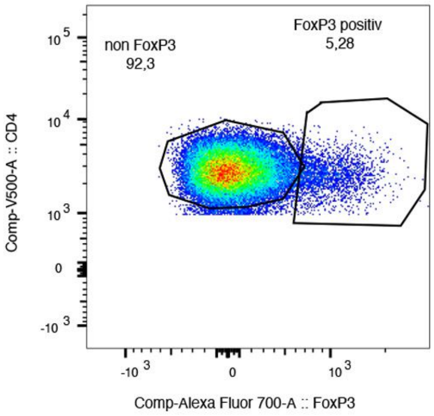

Lito/FBZ M4

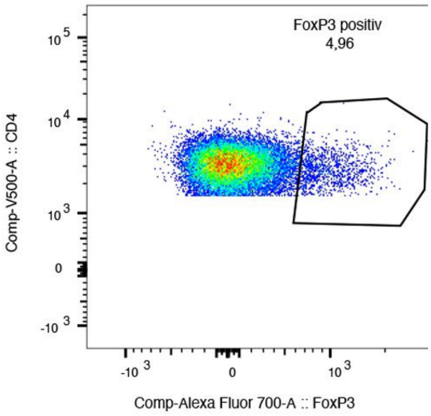

Lito/FBZ M5

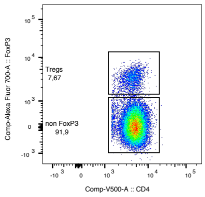

TC_Lito 1.fcs
CD4
24888

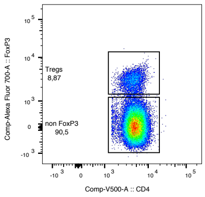

TC_Lito 2.fcs
CD4
38952

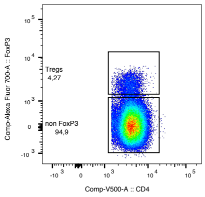

TC_Lito 3.fcs
CD4
73740

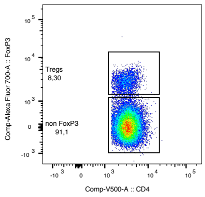

TC_Lito 4.fcs
CD4
31090

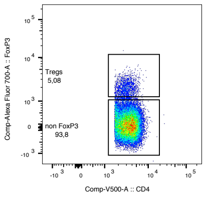

TC_Lito 5.fcs
CD4
24983

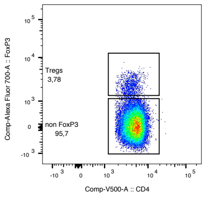

TC_Lito+FBZ 1.fcs
CD4
28179

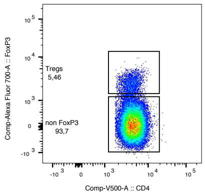

TC_Lito+FBZ 2.fcs
CD4
43040

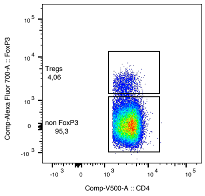

TC_Lito+FBZ 3.fcs
CD4
32355

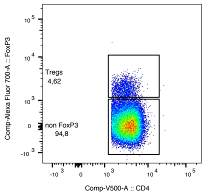

TC_Lito+FBZ 4.fcs
CD4
34791

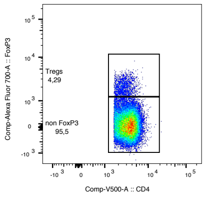

TC_Lito+FBZ 5.fcs
CD4
33517

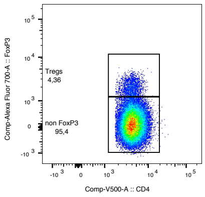

TC_Naiv 1+2.fcs
CD4
44347

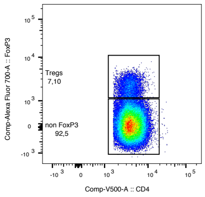

TC_naiv 3.fcs
CD4
75799

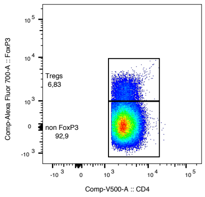

TC_naiv 4+5.fcs
CD4
76941

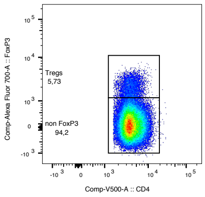

TC_naiv+FBZ 1.fcs
CD4
73184

Dot Plots used for Figure 3E (Treg in the spleen):

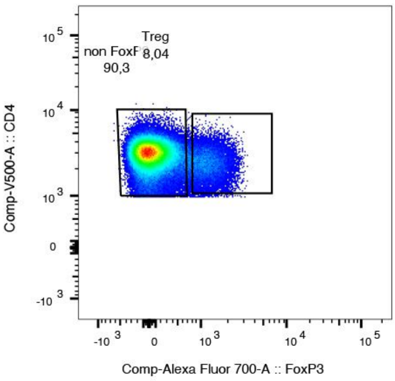

naive M1

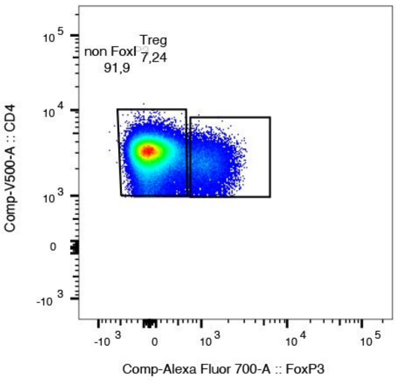

naive M2

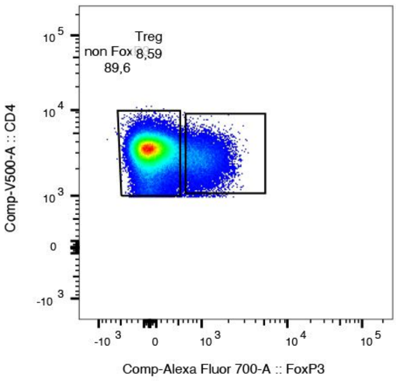

naive M3

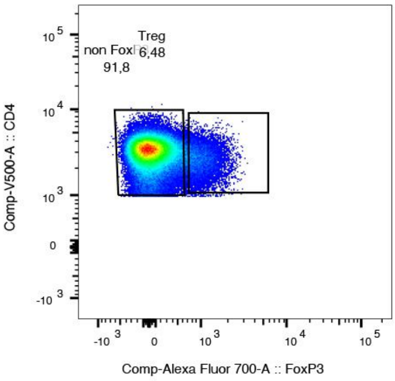

naive M4

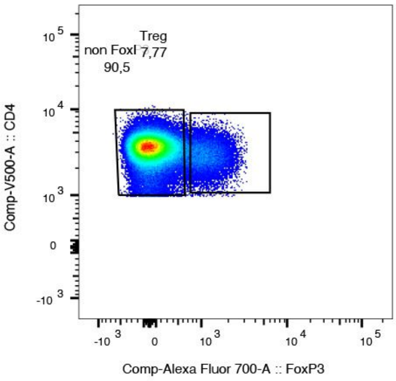

naive M5

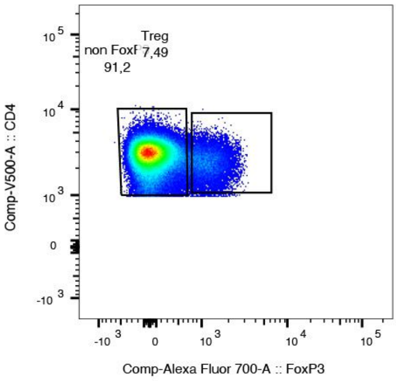

FBZ M1

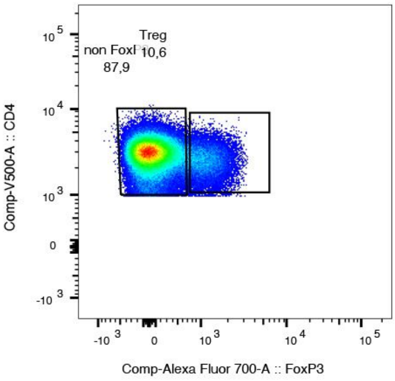

FBZ M2

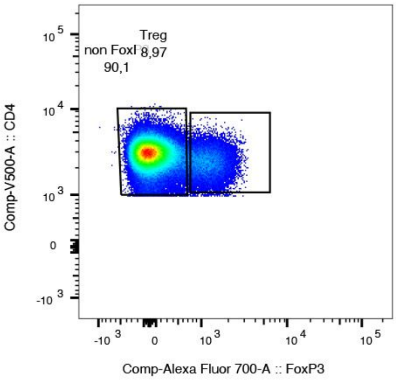

FBZ M3

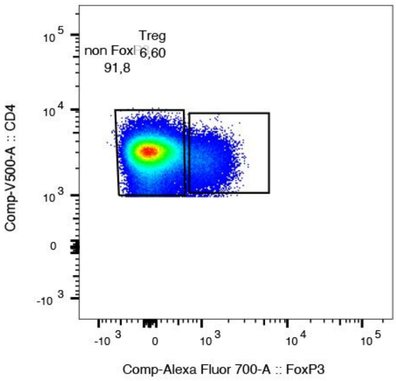

FBZ M4

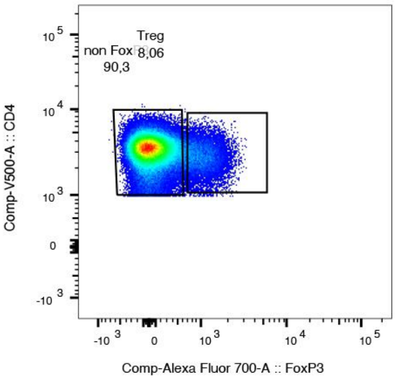

FBZ M5

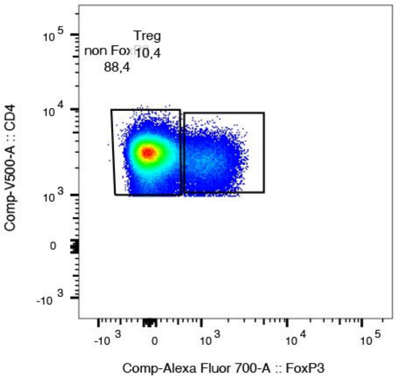

Lito M1

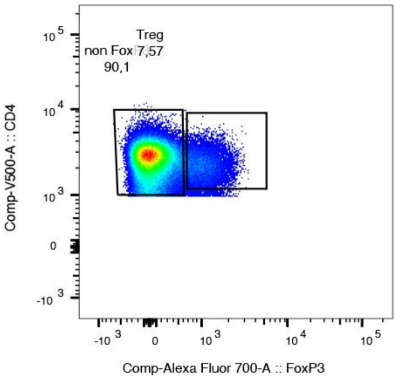

Lito M2

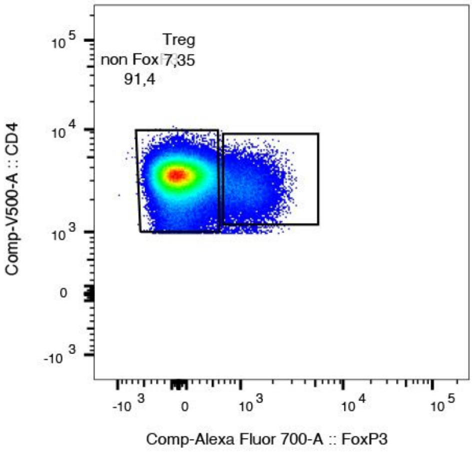

Lito M3

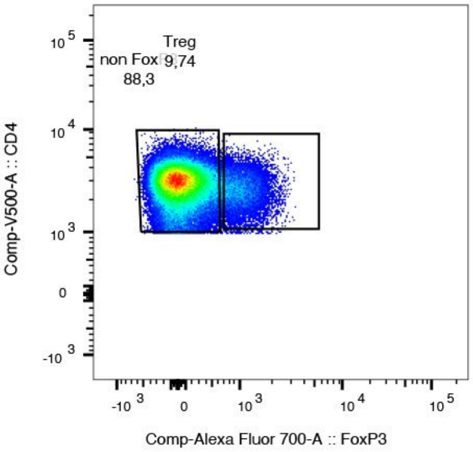

Lito M4

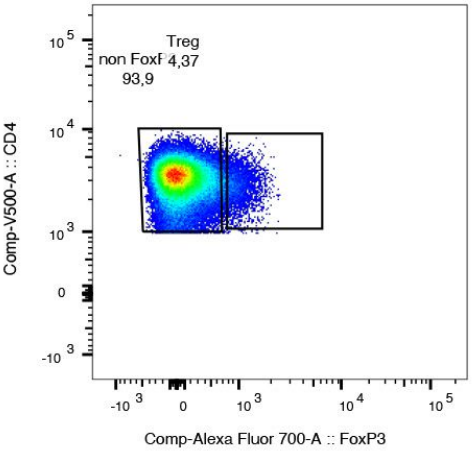

Lito M5

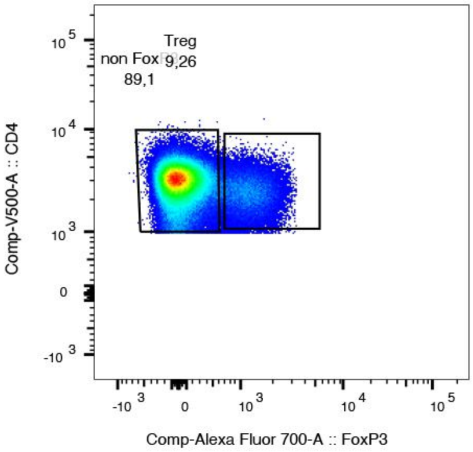

Lito/FBZ M1

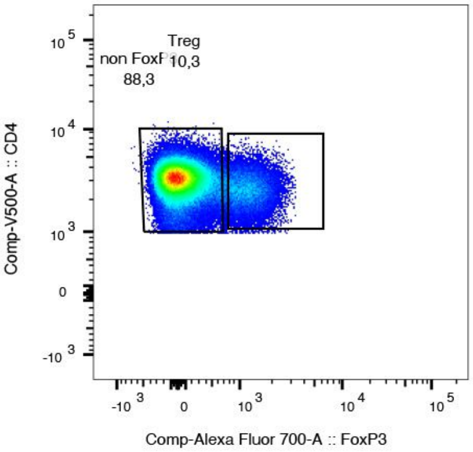

Lito/FBZ M2

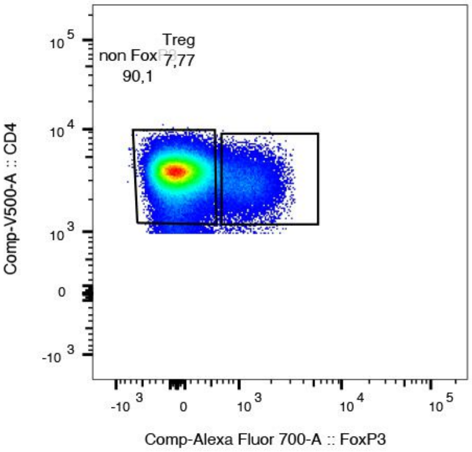

Lito/FBZ M3

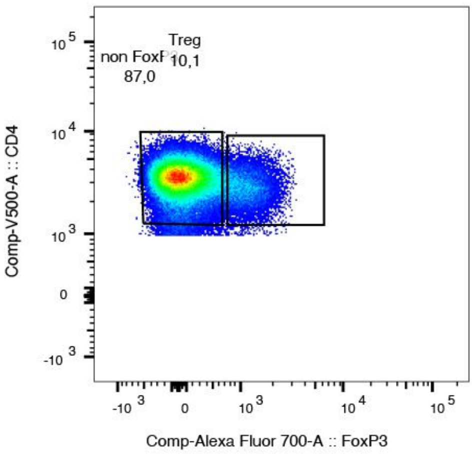

Lito/FBZ M4

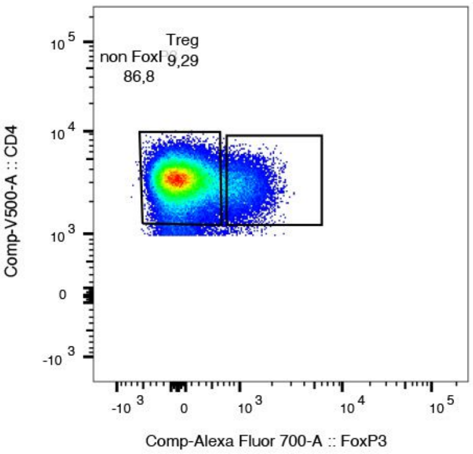

Lito/FBZ M5

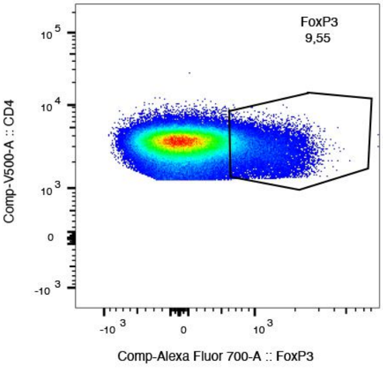

naive M1

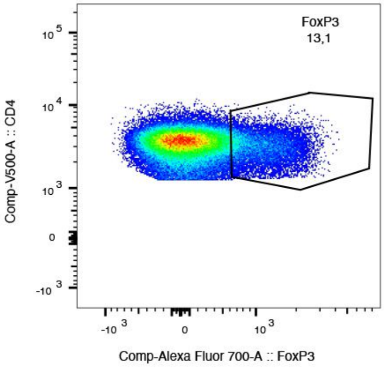

naive M2

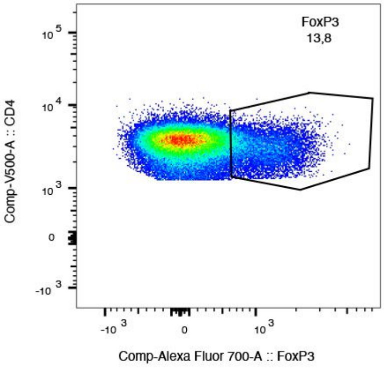

naive M3

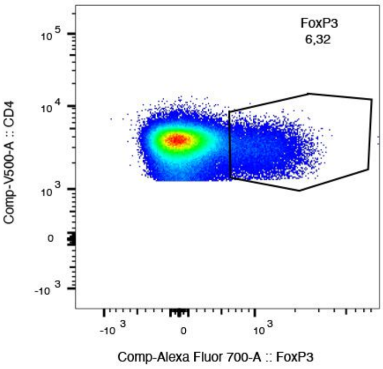

naive M4

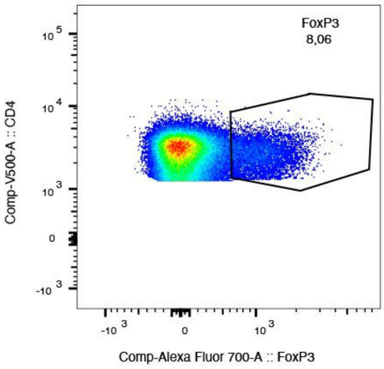

naive M5

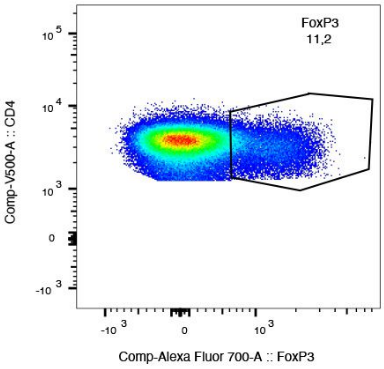

FBZ M1

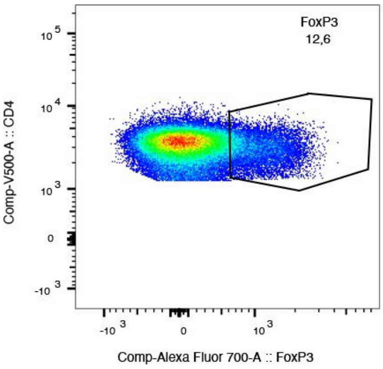

FBZ M2

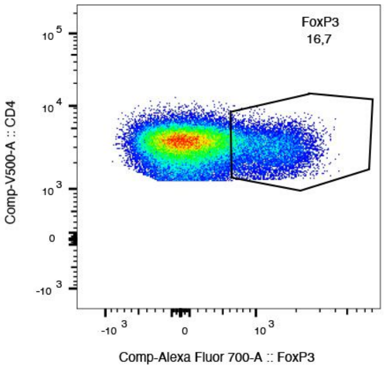

FBZ M3

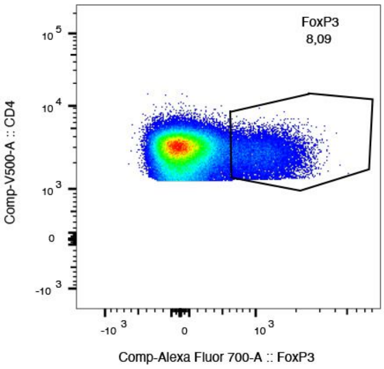

FBZ M4

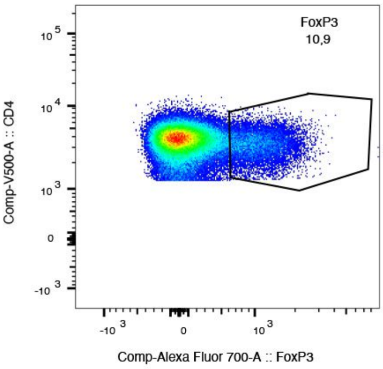

FBZ M5

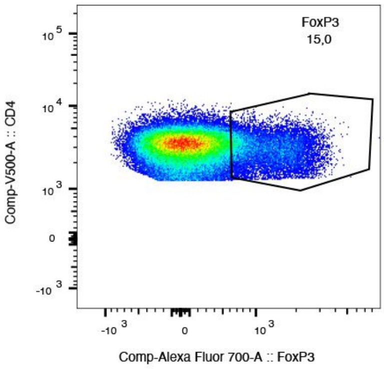

Lito M1

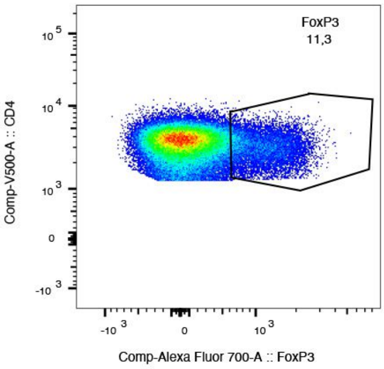

Lito M2

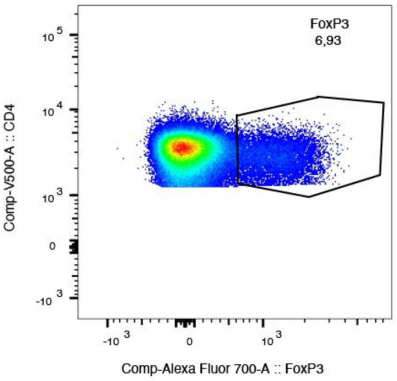

Lito M3

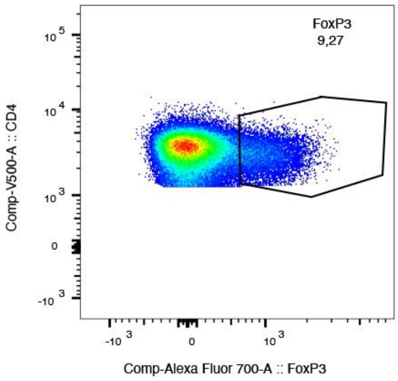

Lito M4

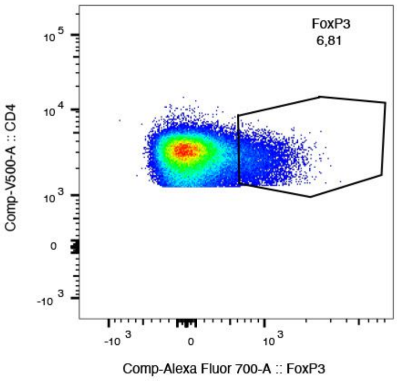

Lito M5

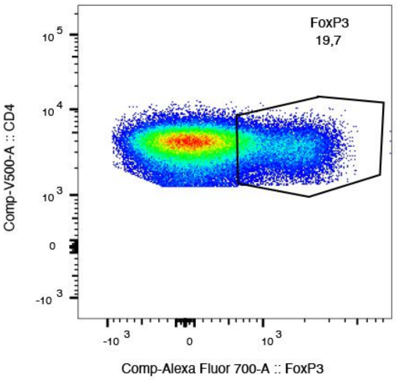

Lito/FBZ M1

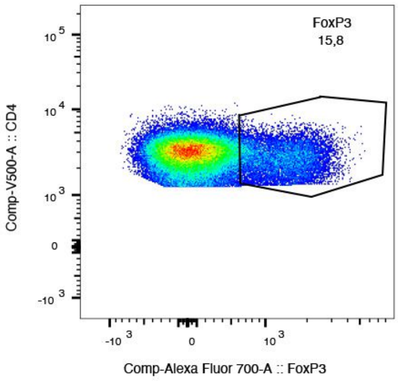

Lito/FBZ M2

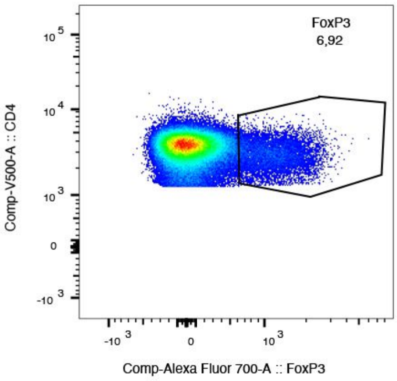

Lito/FBZ M3

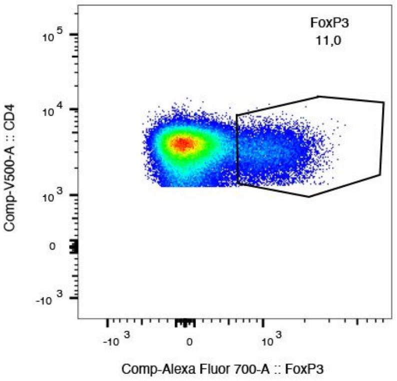

Lito/FBZ M4

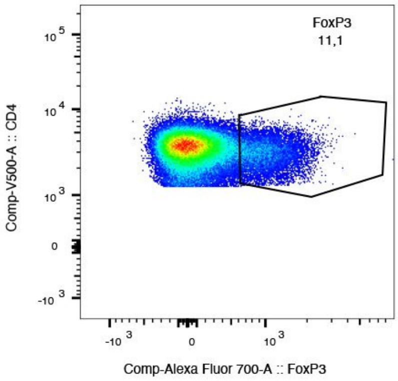

Lito/FBZ M5

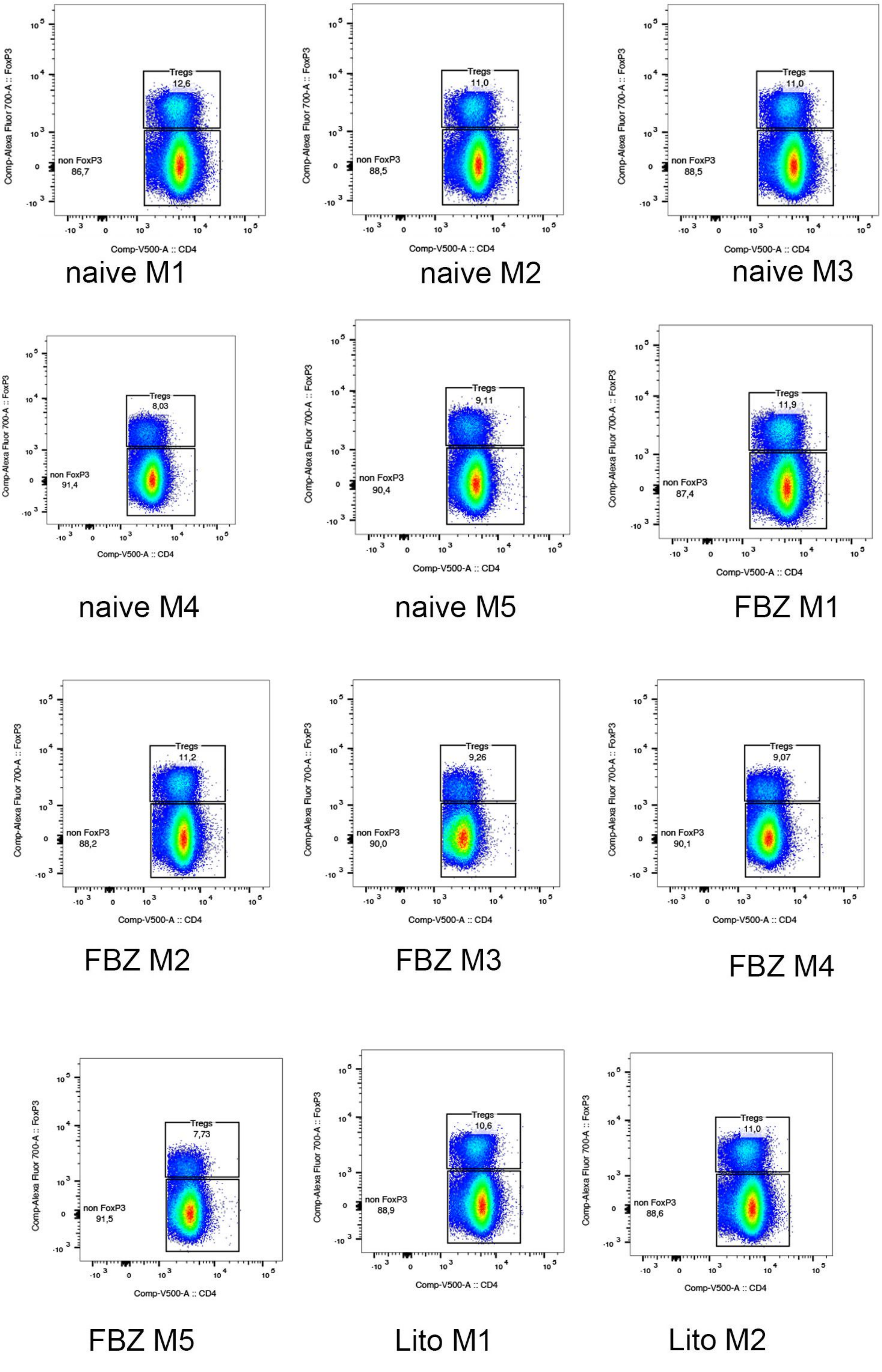

naive M1
Tregs 12,6
non FoxP3 86,7
naive M2
Tregs 11,0
non FoxP3 88,5
naive M3
Tregs 11,0
non FoxP3 88,5
naive M4
Tregs 8,03
non FoxP3 91,4
naive M5
Tregs 9,11
non FoxP3 90,4
FBZ M1
Tregs 11,9
non FoxP3 87,4
FBZ M2
Tregs 11,2
non FoxP3 88,2
FBZ M3
Tregs 9,26
non FoxP3 90,0
FBZ M4
Tregs 9,07
non FoxP3 90,1
FBZ M5
Tregs 7,73
non FoxP3 91,5
Lito M1
Tregs 10,6
non FoxP3 88,9
Lito M2
Tregs 11,0
non FoxP3 88,6
Comp-Alexa Fluor 700-A :: FoxP3
Comp-V500-A :: CD4

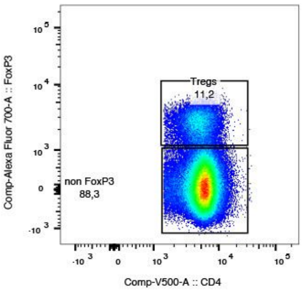

Lito M3

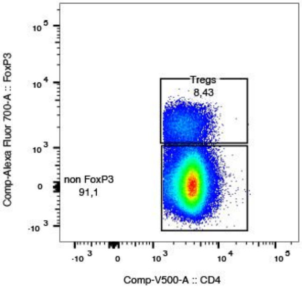

Lito M4

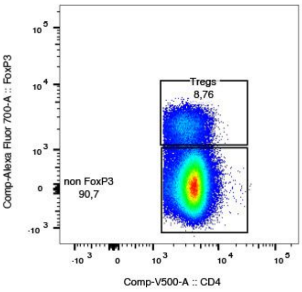

Lito M5

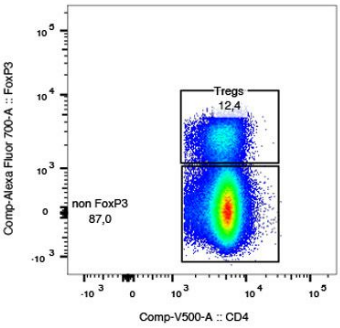

Lito/FBZ M1

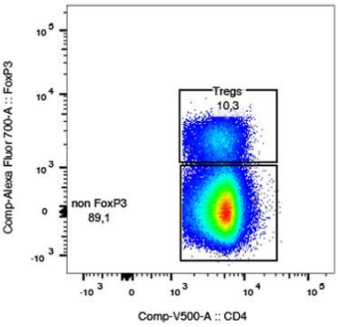

Lito/FBZ M2

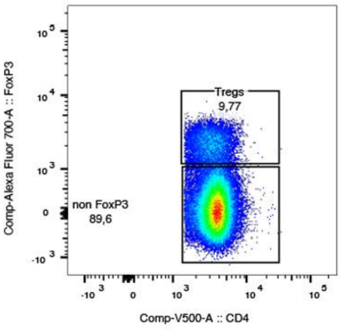

Lito/FBZ M3

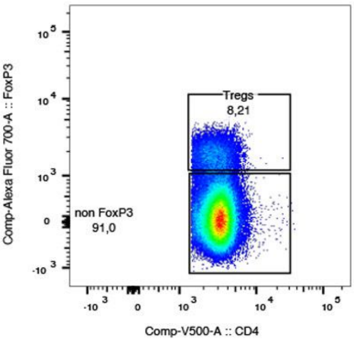

Lito/FBZ M4

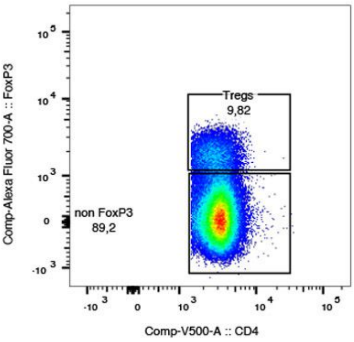

Lito/FBZ M5
